# Study on the correlation between SPISE-IR, METs-IR, TyG and short-term adverse cardiovascular events in patients with heart failure and diabetes mellitus

**DOI:** 10.3389/fnut.2026.1938459

**Published:** 2026-10-16

**Authors:** Haoxuan Deng, Qiyue Zhu, Changjin Lin, Wei Qiu, Yunyan Zhang, Junyi Hua

**Affiliations:** Second Affiliated Hospital, Zhejiang Chinese Medical University, Hangzhou, China

**Keywords:** heart failure, insulin resistance, MACE, prediction model, type 2 diabetes

## Abstract

**Objective:**

Heart failure (HF) patients with type 2 diabetes mellitus (T2DM) are at high risk of short-term major adverse cardiovascular events (MACE). Insulin resistance (IR) is a key shared mechanism, yet simple prognostic markers remain limited. This multicenter retrospective cohort study compared three IR surrogate indices (SPISE-IR, METs-IR, and TyG) for predicting 18-month post-discharge MACE in hospitalized HF-T2DM patients, aiming to identify practical tools for risk stratification.

**Methods:**

A total of 477 hospitalized HF-T2DM patients from two tertiary hospitals in Zhejiang (2018–2024) were enrolled according to unified criteria. Baseline clinical, laboratory, echocardiographic, and medication data were collected to calculate SPISE-IR, METs-IR, and TyG. The primary outcome was 18-month post-discharge composite MACE. Associations were evaluated using Cox regression and RCS, while predictive performance was assessed by ROC and PR curves, DCA, NRI, IDI, and subgroup analyses.

**Results:**

Of 477 patients, 191 (40.04%) experienced MACE over 18 months, including 3 cardiovascular deaths. Patients with MACE were older, had lower LVEF, worse renal function, and higher NT-proBNP (all *p* < 0.05). On multivariable analysis, SPISE-IR (HR 9.31, 95% CI 5.96–14.55, *p* < 0.001) and METs-IR (HR 1.07, 95% CI 1.05–1.09, *p* < 0.001) were associated with MACE, whereas TyG was not (HR 1.19, 95% CI 0.95–1.49, *p* = 0.127). SPISE-IR and METs-IR showed significant nonlinear dose–response relationships and yielded AUCs of 0.790 and 0.801, respectively, versus 0.539 for TyG. Higher SPISE-IR and METs-IR quartiles were associated with progressively higher cumulative event risk (log-rank *p* < 0.001), whereas no significant between-quartile difference was observed for TyG. Adding either index to the baseline model significantly improved NRI and IDI (all *p* < 0.05); SPISE-IR showed numerically larger NRI and IDI improvements than METs-IR. Subgroup analyses showed a consistent SPISE-IR association, whereas METs-IR exhibited effect modification by age, sex, smoking status, and hypertension; TyG remained largely non-significant. Heart failure rehospitalization accounted for 138 of 191 MACE (72.3%). After exclusion of 24 recurrent-angina events, SPISE-IR (HR 10.83, 95% CI 6.71–17.45) and METs-IR (HR 1.07, 95% CI 1.06–1.09) remained associated with the strict MACE endpoint (both *p* < 0.001). After 1,000 bootstrap resamples, optimism-corrected AUCs were 0.818 for the clinical + SPISE-IR model and 0.806 for the clinical + METs-IR model. In center-based external validation, their AUCs were 0.804 and 0.806, respectively.

**Conclusion:**

SPISE-IR and METs-IR are associated with 18-month MACE risk in hospitalized HF-T2DM patients and show better predictive performance than TyG. These indices may provide additional value for risk stratification in this population.

## 1 Introduction

Heart failure (HF) represents the terminal stage of a broad spectrum of cardiovascular diseases and remains a leading cause of morbidity and mortality worldwide (1). A substantial proportion of patients with HF present with concomitant type 2 diabetes mellitus (T2DM), with a reported prevalence of approximately 44% among hospitalized HF populations (2). The coexistence of HF and T2DM is associated with a significantly increased risk of short-term adverse cardiovascular events and worse clinical outcomes (2). T2DM contributes to HF progression through multiple mechanisms. Direct myocardial injury is driven by impaired glucose utilization, lipid accumulation, and mitochondrial dysfunction, leading to energetic deficiency and structural remodeling. In parallel, T2DM accelerates HF through clustering of cardiometabolic comorbidities, including hypertension, coronary artery disease, chronic kidney disease, and obesity (3), which collectively exacerbate ventricular dysfunction.

Insulin resistance (IR), a central feature of T2DM, plays a key role in linking metabolic dysfunction with HF progression. IR promotes chronic inflammation, oxidative stress, and endothelial dysfunction (4), while also impairing mitochondrial function, thereby accelerating myocardial remodeling. In addition, IR is associated with skeletal muscle catabolism and reduced functional capacity, contributing to frailty in HF patients (5).

Patients with HF and T2DM frequently experience rapid clinical deterioration and recurrent decompensation. Early identification of individuals at high short-term risk is therefore critical for optimizing treatment strategies. Risk stratification may support individualized management, including balancing anticoagulation strategies in patients with atrial fibrillation and competing thrombotic and bleeding risks. From a healthcare system perspective, the burden of T2DM-related cardiovascular comorbidities has increased substantially, leading to rising hospitalization demand and resource utilization (6). Early risk identification followed by timely optimization of outpatient therapy, including diuretic adjustment and initiation of sodium-glucose cotransporter-2 inhibitors (SGLT2i), may reduce avoidable hospitalizations and improve healthcare efficiency (7, 8).

Despite advances in biomarker research, reliable tools for short-term risk prediction in HF patients with T2DM remain limited (9). Conventional biomarkers, including BNP, NT-proBNP, and HbA1c, are influenced by age, renal function, and acute physiological stress, limiting their specificity for short-term prognostic assessment (10–12). Therefore, there is an unmet need for simple and routinely available biomarkers for early risk stratification. Recently, surrogate indices of insulin resistance derived from routine clinical parameters, including SPISE-IR, METs-IR, and the TyG index, have gained attention. These indices are non-invasive, cost-effective, and highly reproducible (13, 14). SPISE-IR has been associated with long-term cardiovascular outcomes in metabolic disease and T2DM (15). METs-IR and TyG have both been linked to adverse cardiovascular outcomes and HF prognosis (16, 17).

However, comparative evidence on the predictive performance of SPISE-IR, METs-IR, and TyG in HF patients with T2DM remains limited, particularly for short-term outcomes (18, 19). Head-to-head evaluation within a single cohort is still lacking. Therefore, this study aimed to investigate the associations between SPISE-IR, METs-IR, and TyG and short-term adverse cardiovascular events in HF patients with T2DM, and to compare their predictive performance, providing evidence for simple and clinically applicable risk stratification strategies.

## 2 Methods

### 2.1 Study design and data source

This retrospective cohort study included patients hospitalized for HF and T2DM between January 2018 and June 2024 at Zhejiang Xinhua Hospital, and between January 2020 and June 2024 at Zhejiang Provincial Hospital of Traditional Chinese Medicine. The study was designed to ensure adequate sample size and population representativeness. The date of discharge from the index hospitalization was defined as cohort entry (time zero), and baseline variables were obtained during that hospitalization. Eligibility did not require survival for the full 18-month period after discharge.

### 2.2 Study population

#### 2.2.1 Inclusion criteria

Patients were included if they met all of the following criteria: (1) diagnosis of HF based on clinical symptoms, signs, and objective evidence of cardiac structural and/or functional abnormalities; (2) diagnosis of T2DM according to the American Diabetes Association criteria (2020); (3) New York Heart Association (NYHA) functional classification documented; (4) ascertainable 18-month outcome status, defined as either a MACE occurring within 18 months after discharge or documented MACE-free follow-up through 18 months; and (5) documented anti-heart failure pharmacological therapy. Limited missingness in baseline covariates was not an exclusion criterion and was handled by multiple imputation. Eligibility referred to ascertainment of the 18-month endpoint rather than completion of 18 months of event-free survival. Patients who experienced cardiovascular death within 18 months remained eligible, whereas patients with neither a documented MACE nor confirmation of MACE-free status through 18 months were excluded because their primary endpoint status could not be determined.

#### 2.2.2 Exclusion criteria

Patients were excluded if they met any of the following: (1) NYHA class I; (2) severe hepatic or renal dysfunction; (3) coexisting cardiomyopathy, valvular heart disease, congenital heart disease, pericardial disease, or severe cerebrovascular disease; (4) active malignancy; (5) acute or chronic infections, acute diabetic complications, or other conditions causing stress hyperglycemia; (6) use of glucocorticoids, thyroid hormones, or β-adrenergic agonists affecting glucose metabolism; (7) change in antidiabetic therapy within 1 month prior to admission (Figure 1).

**Figure 1:**
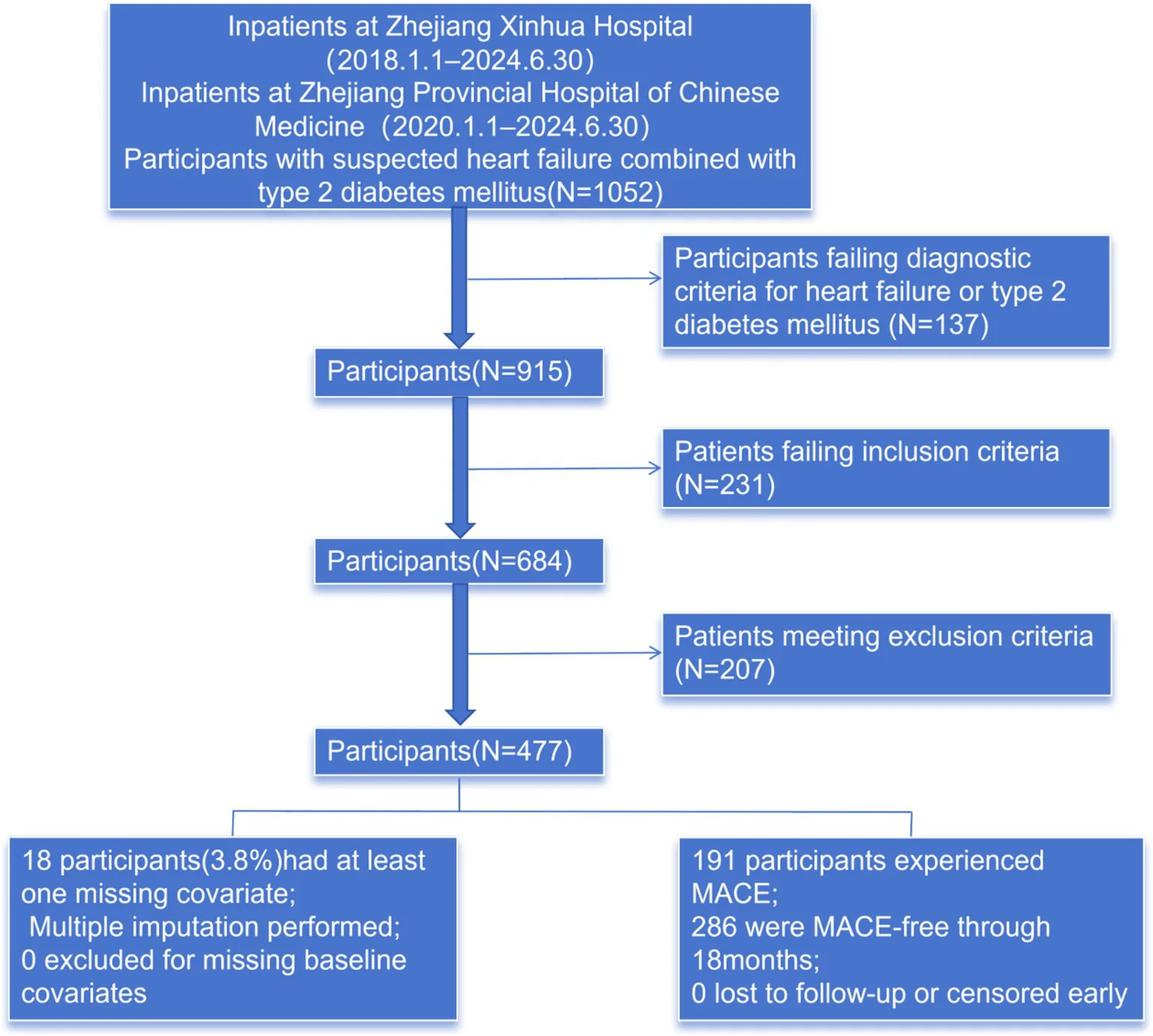
Flowchart of participant screening and enrollment in this retrospective study. A total of 1,052 potentially eligible inpatients were screened. After exclusion of 137 patients who did not meet the diagnostic criteria, 231 who failed other inclusion criteria, and 207 who met prespecified exclusion criteria, 477 participants entered the final analysis. Eighteen participants (3.8%) had at least one missing baseline covariate and were retained using multiple imputation; none were excluded solely for missing baseline variables. During follow-up, 191 participants experienced MACE and 286 remained MACE-free through 18 months, with no loss-to-follow-up censoring before 18 months.

### 2.3 Data collection

Baseline clinical data were extracted from electronic medical records using a standardized data collection form. Variables included demographic characteristics, comorbidities, medication history, laboratory parameters, inflammatory markers, cardiac biomarkers, and echocardiographic measurements. Detailed variable definitions, measurement units, reference ranges, and formulas for the insulin resistance indices are presented in Table 1. SPISE-IR was defined as 10 divided by the conventional SPISE score so that higher values indicate greater insulin resistance. Missingness was assessed for each baseline variable. 18 of the 477 participants (3.8%) had at least one missing baseline covariate value; no participant was excluded solely because of missing baseline covariates.

**Table 1:** Data collection.

Indicator category	Specific indicator	Variable type	Indicator description or calculation method
General clinical data	Age	Continuous variable	Years
	Gender	Categorical variable	Male, Female
	Height	Continuous variable	cm
	Weight	Continuous variable	kg
	Follow-up duration	Continuous variable	Months
	Smoking history	Categorical variable	Yes, No
	Hypertension history	Categorical variable	Yes, No
	Chronic kidney disease history	Categorical variable	Yes, No
	Heart failure classification	Categorical variable	Classified and recorded based on left ventricular ejection fraction (LVEF)
	Myocardial infarction history	Categorical variable	Yes, No
	Previous PCI history	Categorical variable	Yes, No
	Hypoglycemic drug use	Categorical variable	Yes, No
	Antihypertensive drug use	Categorical variable	Yes, No
	NYHA cardiac function classification	Categorical variable	Classified into grade I, II, III, IV according to the NYHA cardiac function classification diagnostic criteria
Prognostic outcome indicators	Short-term adverse cardiovascular events	Categorical variable	Occurred, not occurred
Biochemical indicators	Glycated hemoglobin (HbA1c)	Continuous variable	%
	Fasting plasma glucose (FPG)	Continuous variable	mmol/L
	Triglyceride (TG)	Continuous variable	mmol/L
	Total cholesterol (TC)	Continuous variable	mmol/L
	Low-density lipoprotein cholesterol (LDL-C)	Continuous variable	mmol/L
	High-density lipoprotein cholesterol (HDL-C)	Continuous variable	mmol/L
	Serum creatinine (Scr)	Continuous variable	μmol/L
	Lactate dehydrogenase (LDH)	Continuous variable	U/L
	Albumin (ALB)	Continuous variable	g/L
	Alanine aminotransferase (ALT)	Continuous variable	U/L
	Aspartate aminotransferase (AST)	Continuous variable	U/L
Renal function assessment indicators	Estimated glomerular filtration rate (e-GFR)	Continuous variable	mL·min^−1^·1.73m^−2^
Blood routine and inflammation indicators	Red blood cell count (RBC)	Continuous variable	×10^12^/L
	White blood cell count (WBC)	Continuous variable	×10^9^/L
	Platelet count (PLT)	Continuous variable	×10^9^/L
	Absolute neutrophil count (NEUT#)	Continuous variable	×10^9^/L
	Neutrophil percentage (NEUT%)	Continuous variable	%
	C-reactive protein (CRP)	Continuous variable	mg/L
Myocardial injury markers	N-terminal pro-B-type natriuretic peptide (NT-proBNP)	Continuous variable	pg/mL
	Cardiac troponin (cTn)	Continuous variable	μg/L
	Creatine kinase-MB Isoenzyme (CK-MB)	Continuous variable	U/L
Cardiac ultrasound indicators	Left ventricular ejection fraction (LVEF)	Continuous variable	%
	Left ventricular end-diastolic diameter (LVDD)	Continuous variable	mm
Insulin resistance assessment indicators	SPISE-IR	Continuous variable	SPISE-IR = 10/(600 × HDL^0.185/TG^0.2/BMI^1.338)
	METs-IR	Continuous variable	METs-IR = Ln([2 × G0 + TG0] × BMI)/Ln(HDL-C)
	TyG	Continuous variable	TyG = ln[TG × FPG/2]

HbA1c, glycated hemoglobin; FPG, fasting plasma glucose; TG, triglyceride; TC, total cholesterol; LDL-C, low-density lipoprotein cholesterol; HDL-C, high-density lipoprotein cholesterol; Scr, serum creatinine; LDH, lactate dehydrogenase; ALB, albumin; ALT, alanine aminotransferase; AST, aspartate aminotransferase; e-GFR, estimated glomerular filtration rate; RBC, red blood cell count; WBC, white blood cell count; PLT, platelet count; NEUT#, absolute neutrophil count; NEUT%, neutrophil percentage; CRP, C-reactive protein; NT-proBNP, N-terminal pro-B-type natriuretic peptide; cTn, cardiac troponin; CK-MB, creatine kinase-MB isoenzyme; LVEF, left ventricular ejection fraction; LVDD, left ventricular end-diastolic diameter; BMI, body mass index; Ln, natural logarithm; PCI, percutaneous coronary intervention; NYHA, New York Heart Association.

### 2.4 Outcomes

The primary endpoint was major adverse cardiovascular events (MACE) occurring within 18 months after discharge. MACE was defined as a composite of recurrent angina requiring medical attention, non-fatal myocardial infarction, malignant arrhythmias (including ventricular fibrillation, sustained ventricular tachycardia, and advanced atrioventricular block), heart failure rehospitalization, and cardiovascular death. Time-to-event was defined as the interval from hospital discharge to the first occurrence of MACE or end of follow-up. Patients without events were censored at 18 months.

### 2.5 Statistical analysis

All statistical analyses were performed using SPSS version 27.0. Continuous variables were tested for normality and expressed as mean ± standard deviation or median (interquartile range), as appropriate, while categorical variables were presented as frequencies and percentages. Group comparisons were conducted using Student’s t-test, Mann–Whitney U test, or chi-square test as appropriate. Missing baseline covariates were handled by multiple imputation before calculation of the insulin resistance indices and model fitting. The imputation model included variables used in the primary analyses and the outcome indicator, and estimates were pooled according to Rubin’s rules. Outcome status was not imputed.

Multicollinearity among covariates was assessed using the variance inflation factor (VIF), with VIF < 10 indicating acceptable independence. Associations between SPISE-IR, METs-IR, TyG index, and MACE were evaluated using Cox proportional hazards regression models. Variables with clinical relevance and no significant multicollinearity were entered into multivariate models. Hazard ratios (HRs) and 95% confidence intervals (CIs) were calculated.

The proportional hazards (PH) assumption for each fully adjusted Model 3 Cox model was assessed using tests based on scaled Schoenfeld residuals, both for the insulin resistance index term and globally for the complete model. A two-sided *p* value <0.05 was considered evidence against the PH assumption. When non-proportionality was detected for SPISE-IR, an extended Cox model including an interaction between SPISE-IR and the natural logarithm of follow-up time was fitted as a sensitivity analysis, and time-specific HRs were estimated at 1, 3, 6, 12, and 18 months. Because the TyG term itself did not show statistically significant non-proportionality despite a significant global test, a TyG-specific time interaction was not introduced.

Restricted cubic spline (RCS) analyses were performed within the fully adjusted Model 3 using four knots at the 5th, 35th, 65th, and 95th percentiles of each index. The median value was used as the reference, and both P-overall and P-nonlinearity were reported. Predictive performance was assessed using receiver operating characteristic (ROC) curves with area under the curve (AUC), as well as precision–recall (PR) curves and decision curve analysis (DCA) to evaluate discrimination and clinical utility. Incremental predictive value at 18 months was assessed using the continuous (category-free) net reclassification improvement (NRI) and integrated discrimination improvement (IDI) by comparing the baseline model with models containing each insulin resistance index. Continuous NRI was calculated as the sum of the net proportion of participants with MACE whose predicted risk increased and the net proportion of participants without MACE whose predicted risk decreased after addition of the index; therefore, no risk-category thresholds were used. IDI was calculated as the difference between the new and baseline models in discrimination slopes, defined as the difference in mean predicted risk between participants with and without MACE.

To distinguish the prognostic information provided by the composite insulin resistance indices from that of their individual components, additional Cox models were fitted using each component separately and prespecified component combinations. Model linear predictors were used to calculate 18-month time-dependent ROC curves and inverse-probability-of-censoring-weighted (IPCW) dynamic AUCs. The component-versus-index sensitivity analysis compared the baseline clinical model plus all corresponding components with the same baseline model plus the derived index; differences were defined as the index model minus the component model. For this sensitivity analysis, continuous NRI and IDI were calculated using IPCW, and 95% CIs and two-sided *p* values were obtained from 1,000 bootstrap resamples.

Because several MACE subtypes had fewer than 20 events, separate fully adjusted analyses were not performed. Instead, events were grouped as heart failure rehospitalization or non-heart-failure-rehospitalization MACE for cause-specific Cox analyses, with competing MACE types censored at occurrence. Robustness was further assessed using a strict MACE endpoint excluding recurrent angina, and Models 1–3 were repeated separately for SPISE-IR and METs-IR. The patient with a missing MACE subtype was censored at the recorded event time.

Subgroup analyses were performed using stratified Cox regression according to age, sex, smoking status, hypertension, chronic kidney disease, myocardial infarction history, and HF subtype. Potential interactions were tested, and results were visualized using forest plots. All tests were two-sided, and a *p* value <0.05 was considered statistically significant.

The baseline clinical model and models adding METs-IR, SPISE-IR, or TyG underwent internal validation with 1,000 bootstrap resamples to estimate optimism-corrected AUCs, calibration slopes, and 18-month calibration curves. These validation models were distinct from the Cox adjustment models in Table 2. For center-based external validation, Zhejiang Xinhua Hospital was used for model development after excluding 2018–2019 admissions (*n* = 210; 71 MACE), and Zhejiang Provincial Hospital of Traditional Chinese Medicine was used for validation (*n* = 223; 93 MACE). Model coefficients and baseline survival were fixed without refitting. External performance was assessed using AUC, Brier score, calibration, DCA, continuous NRI, and IDI, with 95% CIs derived from 1,000 paired bootstrap resamples.

**Table 2:** Cox regression analysis.

Variables	Model 1			Model 2			Model 3		
	HR	95%CI	*p*-value	HR	95%CI	*p*-value	HR	95%CI	*p*-value
SPISE-IR	6.96	4.73–10.25	<0.001	8.71	5.81–13.05	<0.001	9.31	5.96–14.55	<0.001
As categorical variables (quartile)									
Q1	1 (reference)			1 (reference)			1 (reference)		
Q2	0.90	0.47–1.69	0.736	0.91	0.48–1.72	0.775	1.00	0.53–1.90	0.998
Q3	4.23	2.56–7.00	<0.001	4.73	2.85–7.85	<0.001	4.71	2.77–7.99	<0.001
Q4	7.12	4.38–11.58	<0.001	7.61	4.68–12.40	<0.001	7.59	4.60–12.54	<0.001
METs-IR	1.07	1.05–1.08	<0.001	1.07	1.06–1.08	<0.001	1.07	1.05–1.09	<0.001
As categorical variables (quartile)									
Q1	1 (reference)			1 (reference)			1 (reference)		
Q2	1.53	0.81–2.88	0.188	1.59	0.84–3.00	0.150	1.48	0.78–2.81	0.235
Q3	5.35	3.09–9.26	<0.001	5.71	3.28–9.92	<0.001	5.82	3.30–10.25	<0.001
Q4	9.00	5.27–15.36	<0.001	9.53	5.58–16.29	<0.001	8.38	4.81–14.59	<0.001
TyG	1.20	0.96–1.49	0.112	1.23	0.98–1.54	0.068	1.19	0.95–1.49	0.127
As categorical variables (quartile)									
Q1	1 (reference)			1 (reference)			1 (reference)		
Q2	1.02	0.67–1.55	0.939	1.03	0.68–1.57	0.895	1.14	0.73–1.77	0.560
Q3	1.30	0.87–1.93	0.202	1.28	0.85–1.90	0.234	1.25	0.83–1.89	0.289
Q4	1.18	0.79–1.77	0.410	1.28	0.85–1.93	0.233	1.25	0.82–1.91	0.301

HR, hazard ratio; 95%CI, 95% confidence interval; *p*-value, statistical significance value; Q1, the first quartile (reference category); Q2, the second quartile; Q3, the third quartile; Q4, the fourth quartile.

Model 1 was unadjusted. Model 2 was adjusted for age and sex. Model 3 was adjusted for age, sex, smoking history, hypertension, chronic kidney disease, NYHA functional class, history of myocardial infarction, LVEF, LDH, albumin, eGFR, CK-MB, NT-proBNP, cardiac troponin I, red blood cell count, platelet count, neutrophil percentage, and C-reactive protein. SPISE-IR, METs-IR, and TyG were entered separately into independent Cox regression models and were not included simultaneously.

Center-related heterogeneity was evaluated by adding study center and center-by-index interactions to the fully adjusted Cox models and by fitting center-specific models using the Model 3 covariates. Enrollment-period differences were addressed by excluding Xinhua Hospital admissions from 2018 to 2019 and, separately, restricting both centers to 2020–2024. A two-sided interaction *p* < 0.05 indicated significant heterogeneity.

## 3 Results

### 3.1 Baseline characteristics

A total of 477 patients with HF and T2DM were included, with 191 (40.04%) experiencing MACE during 18-month follow-up. Compared with the non-MACE group (Table 3), patients in the MACE group were older, had lower LVEF, and exhibited higher prevalence of chronic kidney disease and advanced HF status (higher proportion of NYHA class III–IV and HFrEF phenotype). In addition, NT-proBNP, creatinine, LDH, CK-MB, and RBC levels were significantly higher, while HDL-C and eGFR were significantly lower (all *p* < 0.05). Notably, HbA1c levels were lower in the MACE group (*p* = 0.004). Among the 477 included participants, 18 (3.8%) had at least one missing baseline covariate and were retained after multiple imputation; therefore, no participant was excluded from the analytic cohort because of missing baseline variables.

**Table 3:** Baseline characteristics of the MACE group and the non-MACE group.

Variables	Total (*n* = 477)	Without-MACE (*n* = 286)	With MACE (*n* = 191)	Statistic	*p*
Age (years)	78.00 (70.00, 85.00)	77.00 (69.00, 83.00)	81.00 (72.00, 86.00)	Wilcox.	<0.001
LVEF (%)	57.00 (49.00, 64.00)	58.00 (50.00, 65.47)	56.00 (49.00, 62.00)	Wilcox.	0.010
LVDD (mm)	50.00 (46.00, 54.00)	49.15 (46.00, 54.00)	50.00 (46.85, 54.00)	Wilcox.	0.061
HbA1c (%)	6.90 (6.30, 7.80)	7.20 (6.30, 7.97)	6.70 (6.20, 7.55)	Wilcox.	0.004
FPG (mmol/L)	6.72 (4.81, 9.58)	6.79 (5.18, 9.10)	6.59 (4.09, 10.34)	Wilcox.	0.617
TC (mmol/L)	3.79 (3.06, 4.54)	3.95 (3.16, 4.54)	3.56 (2.84, 4.55)	Wilcox.	0.064
LDL-C (mmol/L)	1.77 (1.32, 2.45)	1.84 (1.37, 2.43)	1.71 (1.29, 2.54)	Wilcox.	0.329
TG (mmol/L)	1.12 (0.76, 1.89)	1.07 (0.77, 1.59)	1.21 (0.72, 2.38)	Wilcox.	0.253
HDL-C (mmol/L)	0.97 (0.57, 1.53)	1.04 (0.65, 1.45)	0.73 (0.50, 1.69)	Wilcox.	0.152
Cr (μmol/L)	90.00 (69.80, 125.00)	86.00 (66.00, 110.83)	101.10 (77.40, 138.25)	Wilcox.	<0.001
LDH (U/L)	197.00 (165.00, 237.00)	192.50 (163.25, 232.75)	207.00 (177.00, 242.00)	Wilcox.	0.025
ALB (g/L)	34.80 (31.70, 38.10)	34.90 (32.12, 38.10)	34.30 (31.10, 37.95)	Wilcox.	0.160
ALT (U/L)	18.00 (12.00, 29.00)	18.00 (12.00, 28.00)	17.00 (12.00, 29.50)	Wilcox.	0.909
AST (U/L)	22.00 (17.00, 31.00)	22.00 (16.00, 30.00)	22.00 (18.00, 31.50)	Wilcox.	0.332
eGFR (mL·min^−1^·1.73 m^−2^)	66.20 (47.40, 90.84)	73.31 (52.96, 97.88)	56.10 (42.93, 74.90)	Wilcox.	<0.001
CK-MB (U/L)	12.60 (6.58, 18.20)	12.25 (6.40, 17.05)	13.40 (7.05, 19.65)	Wilcox.	0.028
NT-proBNP (pg/mL)	2387.00 (1238.00, 5364.00)	2022.50 (996.00, 3909.75)	3187.00 (1682.50, 7166.00)	Wilcox.	<0.001
TnI (μg/L)	0.02 (0.01, 0.06)	0.02 (0.01, 0.06)	0.02 (0.01, 0.06)	Wilcox.	0.549
RBC, ×10^12^/L	4.03 ± 0.76	4.10 ± 0.79	3.93 ± 0.72	Welch t	0.011
WBC, ×10^9^/L	6.60 (5.10, 8.20)	6.65 (5.20, 8.20)	6.50 (5.00, 8.45)	Wilcox.	0.706
PLT, ×10^9^/L	175.00 (139.00, 218.00)	175.50 (145.00, 218.75)	170.00 (130.50, 217.00)	Wilcox.	0.192
ANC, ×10^9^/L	4.40 (3.20, 6.00)	4.30 (3.30, 5.97)	4.50 (3.20, 5.95)	Wilcox.	0.962
NEUT (%)	69.23 (60.40, 76.90)	69.25 (60.07, 75.80)	69.23 (60.75, 77.90)	Wilcox.	0.610
CRP (mg/L)	5.00 (1.70, 16.28)	4.91 (1.50, 15.03)	5.68 (2.07, 19.05)	Wilcox.	0.086
Height (cm)	163.00 (156.00, 168.00)	165.00 (156.00, 170.00)	160.00 (155.50, 167.00)	Wilcox.	0.002
Weight (kg)	64.70 (58.00, 72.00)	62.00 (55.00, 70.00)	68.00 (62.00, 75.00)	Wilcox.	<0.001
BMI (kg/m^2^)	23.64 (21.89, 25.58)	24.00 (21.51, 25.17)	23.13 (21.96, 32.68)	Wilcox.	0.082
SPISE	8.18 (7.13, 9.27)	8.79 (8.03, 10.00)	7.20 (6.29, 7.91)	Wilcox.	<0.001
SPISE-IR	1.22 (1.08, 1.40)	1.14 (1.00, 1.25)	1.39 (1.26, 1.59)	Wilcox.	<0.001
METS-IR	39.99 (36.28, 44.73)	38.06 (34.16, 40.67)	44.20 (40.72, 49.55)	Wilcox.	<0.001
TyG	8.76 (8.34, 9.22)	8.72 (8.34, 9.20)	8.79 (8.39, 9.25)	Wilcox.	0.144
Gender				χ^2^	0.253
Male, *n* (%)	250 (52.4%)	156 (54.5%)	94 (49.2%)		
Female, *n* (%)	227 (47.6%)	130 (45.5%)	97 (50.8%)		
Smoking				*χ* ^2^	0.990
Non-smoking, *n* (%)	327 (68.6%)	196 (68.5%)	131 (68.6%)		
Smoking, *n* (%)	150 (31.4%)	90 (31.5%)	60 (31.4%)		
Hypertension				*χ* ^2^	0.156
Without hypertension, *n* (%)	103 (21.6%)	68 (23.8%)	35 (18.3%)		
With hypertension, *n* (%)	374 (78.4%)	218 (76.2%)	156 (81.7%)		
CKD				*χ* ^2^	0.001
Without CKD, *n* (%)	353 (74.0%)	227 (79.4%)	126 (66.0%)		
With CKD, *n* (%)	124 (26.0%)	59 (20.6%)	65 (34.0%)		
Heart failure type				*χ* ^2^	0.002
HFrEF, *n* (%)	168 (35.2%)	83 (29.0%)	85 (44.5%)		
HFmrEF, *n* (%)	78 (16.4%)	48 (16.8%)	30 (15.7%)		
HFpEF, *n* (%)	231 (48.4%)	155 (54.2%)	76 (39.8%)		
NYHA functional class				*χ* ^2^	<0.001
NYHA II, *n* (%)	129 (27.0%)	99 (34.6%)	30 (15.7%)		
NYHA III, *n* (%)	249 (52.2%)	151 (52.8%)	98 (51.3%)		
NYHA IV, *n* (%)	99 (20.8%)	36 (12.6%)	63 (33.0%)		
Previous myocardial infarction				*χ* ^2^	0.552
Without previous MI, *n* (%)	398 (83.4%)	241 (84.3%)	157 (82.2%)		
With previous MI, *n* (%)	79 (16.6%)	45 (15.7%)	34 (17.8%)		
Previous PCI				*χ* ^2^	0.825
Without previous PCI, *n* (%)	347 (72.7%)	207 (72.4%)	140 (73.3%)		
With previous PCI, *n* (%)	130 (27.3%)	79 (27.6%)	51 (26.7%)		
Diabetes				*χ* ^2^	<0.001
Yes, *n* (%)	477 (100.0%)	286 (100.0%)	191 (100.0%)		
Antidiabetic agents				*χ* ^2^	0.017
Without antidiabetic agents, *n* (%)	70 (14.7%)	51 (17.8%)	19 (9.9%)		
With antidiabetic agents, *n* (%)	407 (85.3%)	235 (82.2%)	172 (90.1%)		
Antihypertensive agents				*χ* ^2^	0.017
Without antihypertensive agents, *n* (%)	120 (25.2%)	83 (29.0%)	37 (19.4%)		
With antihypertensive agents, *n* (%)	357 (74.8%)	203 (71.0%)	154 (80.6%)		
Hospital				*χ* ^2^	0.488
Zhejiang Provincial TCM Hospital, *n* (%)	223 (46.8%)	130 (45.5%)	93 (48.7%)		
Zhejiang Xinhua Hospital, *n* (%)	254 (53.2%)	156 (54.5%)	98 (51.3%)		

MACE, major adverse cardiovascular events; WBC, white blood cell count; LVEF, left ventricular ejection fraction; LVDD, left ventricular end-diastolic diameter; HbA1c, glycated hemoglobin; FPG, fasting plasma glucose; TC, total cholesterol; LDL-C, low-density lipoprotein cholesterol; TG, triglyceride; HDL-C, high-density lipoprotein cholesterol; Cr, serum creatinine; LDH, lactate dehydrogenase; ALB, albumin; ALT, alanine aminotransferase; AST, aspartate aminotransferase; eGFR, estimated glomerular filtration rate; CK-MB, creatine kinase-MB isoenzyme; NT-proBNP, N-terminal pro-B-type natriuretic peptide; TnI, cardiac troponin I; RBC, red blood cell count; PLT, platelet count; ANC, absolute neutrophil count; NEUT, neutrophil percentage; CRP, C-reactive protein; BMI, body mass index; CKD, chronic kidney disease; HF, heart failure; HFrEF, heart failure with reduced ejection fraction; HFmrEF, heart failure with mildly reduced ejection fraction; HFpEF, heart failure with preserved ejection fraction; NYHA, New York Heart Association cardiac function classification; MI, myocardial infarction; PCI, percutaneous coronary intervention.

Across insulin resistance index strata (SPISE-IR, METs-IR, and TyG), higher SPISE-IR and METs-IR levels were consistently associated with worse metabolic status, including higher BMI, triglycerides, impaired renal function, and more severe cardiac dysfunction (lower LVEF and higher NT-proBNP). These two indices also showed a clear graded increase in MACE incidence across quartiles (Supplementary Tables S1–S3). In contrast, TyG showed no significant association with MACE incidence across quartiles.

### 3.2 Multivariable Cox regression analysis

After exclusion of variables with significant multicollinearity (Supplementary Table S4), Cox regression was performed. In Model 1 (unadjusted; Table 2), SPISE-IR (HR = 6.96, 95% CI: 4.73–10.25, *p* < 0.001) and METs-IR (HR = 1.07, 95% CI: 1.05–1.08, *p* < 0.001) were significantly associated with MACE risk, whereas TyG was not (HR = 1.20, 95% CI: 0.96–1.49, *p* = 0.112).

After full adjustment (Model 3; Table 2), SPISE-IR (HR = 9.31, 95% CI: 5.96–14.55, *p* < 0.001) and METs-IR (HR = 1.07, 95% CI: 1.05–1.09, *p* < 0.001) remained independently associated with MACE, whereas TyG remained non-significant (HR = 1.19, 95% CI: 0.95–1.49, *p* = 0.127). Age (HR > 1), NYHA class III–IV (HR increased progressively vs. class II), lower LVEF (HR = 0.98, 95% CI: 0.97–0.99), and lower eGFR (HR = 0.99, 95% CI: 0.98–0.99) were also independently associated with MACE.

Tests based on scaled Schoenfeld residuals showed evidence of non-proportionality for SPISE-IR (index-specific chi-square = 6.903, *p* = 0.009; global chi-square = 33.787, *p* = 0.028). The METs-IR model satisfied the PH assumption (index-specific chi-square = 0.371, *p* = 0.542; global chi-square = 30.003, *p* = 0.070). For TyG, the index-specific test was not significant (chi-square = 3.481, *p* = 0.062), although the global test indicated model-level non-proportionality (chi-square = 34.361, *p* = 0.024). In the extended SPISE-IR model, the SPISE-IR x log (time) interaction was significant (coefficient = 0.769, HR = 2.157, *p* = 0.010), confirming that the association varied over follow-up. The estimated time-specific HRs were 2.40 (95% CI: 0.76–7.50) at 1 month, 5.57 (95% CI: 3.05–10.17) at 3 months, 9.49 (95% CI: 6.12–14.74) at 6 months, 16.17 (95% CI: 8.92–29.32) at 12 months, and 22.09 (95% CI: 10.17–47.94) at 18 months (Tables 4, 5). Accordingly, the constant Model 3 HR for SPISE-IR should be interpreted as an average association over follow-up rather than a time-invariant effect.

**Table 4:** Proportional hazards assumption tests for the fully adjusted Model 3 Cox models.

Index	Test term	Chi-square	*p* value
SPISE-IR	SPISE-IR	6.903	0.009
SPISE-IR	GLOBAL	33.787	0.028
METs-IR	METs-IR	0.371	0.542
METs-IR	GLOBAL	30.003	0.070
TyG	TyG	3.481	0.062
TyG	GLOBAL	34.361	0.024

The proportional hazards assumption was assessed using scaled Schoenfeld residuals. GLOBAL refers to the global test for the complete multivariable model. A *p* value <0.05 indicates evidence against the proportional hazards assumption.

**Table 5:** Extended Cox model with the SPISE-IR x log (time) interaction and time-specific hazard ratios.

Model component or time	Coefficient	HR	95% CI	*p* value
SPISE-IR fixed effect	0.874	2.396	–	0.134
SPISE-IR x log (time)	0.769	2.157	–	0.010
Time-specific estimates				
1 month	–	2.40	0.76–7.50	–
3 months	–	5.57	3.05–10.17	–
6 months	–	9.49	6.12–14.74	–
12 months	–	16.17	8.92–29.32	–
18 months	–	22.09	10.17–47.94	–

HR, hazard ratio; CI, confidence interval; MACE, major adverse cardiovascular events.

The fixed-effect HR corresponds to 1 month of follow-up because log (1) = 0. A significant time-interaction term indicates that the association between SPISE-IR and MACE varies over follow-up.

### 3.3 MACE components and sensitivity analyses

Among 191 patients with MACE, heart failure rehospitalization was the predominant component (*n* = 138, 72.3%), followed by recurrent angina (*n* = 24, 12.6%), malignant arrhythmia (*n* = 13, 6.8%), nonfatal myocardial infarction (*n* = 12, 6.3%), and cardiovascular death (*n* = 3, 1.6%); one event was unclassified (Table 6). In grouped cause-specific analyses, SPISE-IR and METs-IR were associated with both heart failure rehospitalization and non-heart-failure-rehospitalization MACE, whereas TyG was not (Table 7; Figure 2). After excluding recurrent angina and censoring the unclassified event, 166 patients met the strict MACE endpoint. The associations remained significant for SPISE-IR (HR = 10.83, 95% CI: 6.71–17.45) and METs-IR (HR = 1.07, 95% CI: 1.06–1.09; both *p* < 0.001), supporting the robustness of the primary findings (Tables 8–10).

**Table 6:** Eighteen-month time-dependent AUCs for METs-IR and its component models.

Model	18-month AUC (95% CI)
METs-IR	0.800 (0.757–0.843)
BMI	0.541 (0.483–0.598)
HDL-C	0.541 (0.483–0.599)
Triglycerides	0.528 (0.471–0.586)
Fasting plasma glucose	0.481 (0.424–0.538)
BMI + HDL-C	0.751 (0.706–0.797)
HDL-C + triglycerides	0.572 (0.518–0.627)
BMI + triglycerides	0.697 (0.645–0.749)
BMI + fasting plasma glucose	0.617 (0.564–0.670)
HDL-C + fasting plasma glucose	0.511 (0.456–0.566)
Triglycerides+fasting plasma glucose	0.591 (0.538–0.645)
BMI + HDL-C + triglycerides	0.790 (0.746–0.833)
BMI + HDL-C + fasting plasma glucose	0.760 (0.716–0.805)
BMI + triglycerides+fasting plasma glucose	0.722 (0.673–0.772)
HDL-C + triglycerides+fasting plasma glucose	0.589 (0.535–0.642)
BMI + HDL-C + triglycerides+fasting plasma glucose	0.792 (0.748–0.836)

AUC, area under curve; 95% CI, 95% confidence interval.

**Table 7:** Eighteen-month time-dependent AUCs for TyG and its component models.

Model	18-month AUC (95% CI)
TyG	0.536 (0.482–0.590)
Triglycerides	0.528 (0.471–0.586)
Fasting plasma glucose	0.481 (0.424–0.538)
Triglycerides+fasting plasma glucose	0.591 (0.538–0.645)

AUC, area under curve; 95% CI, 95% confidence interval.

**Figure 2:**
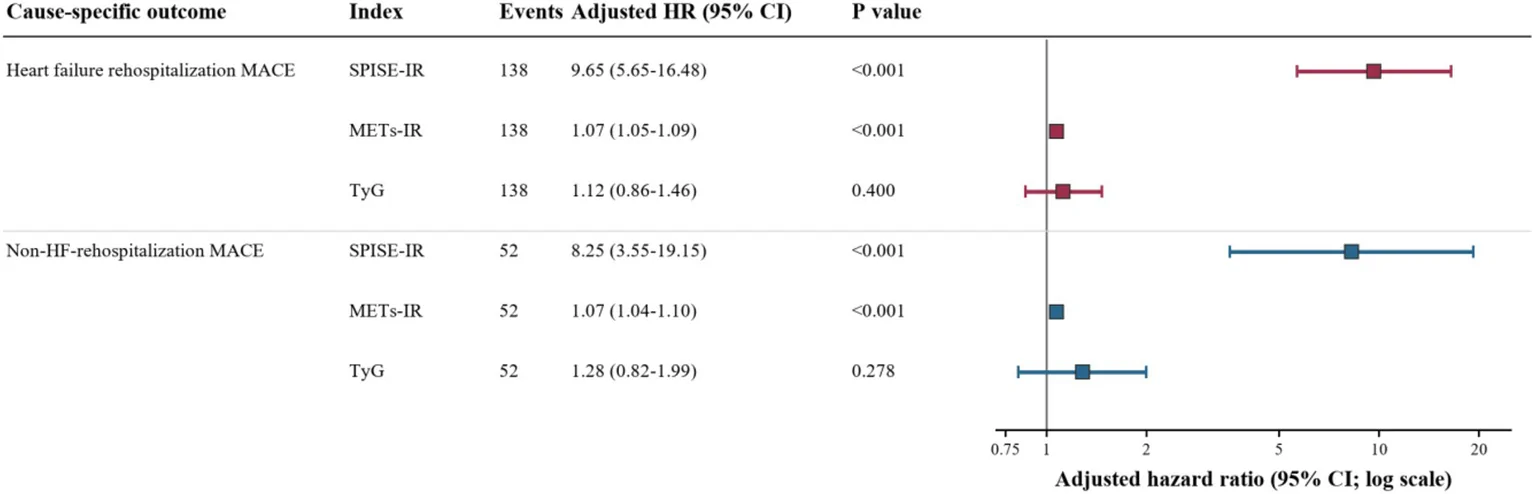
Fully adjusted cause-specific Cox analyses for grouped MACE subtypes. Adjusted HRs and 95% CIs are shown on a logarithmic scale. Non-heart-failure-rehospitalization MACE comprised recurrent angina, nonfatal myocardial infarction, malignant arrhythmia, and cardiovascular death. Other MACE types were censored at their time of occurrence.

**Table 8:** Comparison of component-based and composite-index models at 18 months.

Index	Component model/index model	AUCs	Delta AUC (95% CI), *p*	NRI (95% CI), *p*	IDI (95% CI), *p*
SPISE-IR	Baseline+BMI + HDL-C + TG/Baseline+SPISE-IR	0.853/0.846	−0.006 (−0.015 to 0.002), 0.140	−0.114 (−0.463 to 0.246), 0.522	−0.011 (−0.031 to 0.004), 0.128
METs-IR	Baseline+BMI + HDL-C + TG + FPG/Baseline+METs-IR	0.853/0.837	−0.017 (−0.042 to 0.004), 0.122	−0.481 (−0.668 to 0.173), 0.208	−0.053 (−0.095 to 0.003), 0.072
TyG	Baseline+TG + FPG/Baseline+TyG	0.763/0.748	−0.016 (−0.029 to −0.002), 0.032	−0.282 (−0.568 to −0.003), 0.048	−0.017 (−0.038 to −0.004), 0.012

AUC, area under the curve; BMI, body mass index; HDL-C, high-density lipoprotein cholesterol; TG, triglycerides; FPG, fasting plasma glucose; NRI, net reclassification improvement; IDI, integrated discrimination improvement.

Comparisons are expressed as the index model minus the component model. Continuous NRI and IDI were estimated using IPCW with 1,000 bootstrap resamples for 95% CIs and two-sided *p* values.

**Table 9:** Composition of the 18-month MACE endpoint.

MACE component	Definition	Events, *n*	% of cohort	% of MACE
Recurrent angina	Required medical evaluation or treatment	24	5.0	12.6
Nonfatal myocardial infarction	Nonfatal myocardial infarction	12	2.5	6.3
Malignant arrhythmia	Ventricular fibrillation, sustained ventricular tachycardia, or advanced atrioventricular block	13	2.7	6.8
Heart failure rehospitalization	Rehospitalization for heart failure	138	28.9	72.3
Cardiovascular death	Death from a cardiovascular cause	3	0.6	1.6
Unclassified	MACE recorded but event subtype missing	1	0.2	0.5

Percentages of the cohort use *n* = 477; percentages of MACE use *n* = 191. The five classified components total 190 events; one additional MACE had a missing subtype.

**Table 10:** Fully adjusted cause-specific Cox analyses for grouped MACE subtypes.

Index	Cause-specific outcome	Events, *n*	Adjusted HR (95% CI)	*p* value
SPISE-IR	Heart failure rehospitalization MACE	138	9.65 (5.65–16.48)	<0.001
SPISE-IR	Non-heart-failure-rehospitalization MACE	52	8.25 (3.55–19.15)	<0.001
METs-IR	Heart failure rehospitalization MACE	138	1.07 (1.05–1.09)	<0.001
METs-IR	Non-heart-failure-rehospitalization MACE	52	1.07 (1.04–1.10)	<0.001
TyG	Heart failure rehospitalization MACE	138	1.12 (0.86–1.46)	0.400
TyG	Non-heart-failure-rehospitalization MACE	52	1.28 (0.82–1.99)	0.278

Non-heart-failure-rehospitalization MACE comprised recurrent angina, nonfatal myocardial infarction, malignant arrhythmia, and cardiovascular death. Other MACE types were censored at their time of occurrence. Models used the prespecified full covariate adjustment.

### 3.4 Dose–response relationships

Fully adjusted RCS models used four knots at the 5th, 35th, 65th, and 95th percentiles, with the median of each index as the reference (Table 11; Figure 3). SPISE-IR and METs-IR showed significant overall and nonlinear associations with MACE (both P-overall and P-nonlinearity < 0.001), with risk increasing more steeply above their reference values. TyG showed neither a significant overall association (*p*-overall = 0.264) nor evidence of nonlinearity (*p*-nonlinearity = 0.432).

**Table 11:** RCS knot locations, reference values, and statistical tests.

Index	5th percentile	35th percentile	65th percentile	95th percentile	Reference median	*p*-overall	*p*-nonlinearity
SPISE-IR	0.835	1.138	1.328	1.824	1.223	<0.001	<0.001
METs-IR	29.747	38.091	42.124	55.103	39.991	<0.001	<0.001
TyG	7.892	8.501	9.008	9.935	8.755	0.264	0.432

RCS, restricted cubic spline.

All RCS models were adjusted using the Model 3 covariate set. Four knots were placed at the 5th, 35th, 65th, and 95th percentiles, and the median was used as the reference.

**Figure 3:**
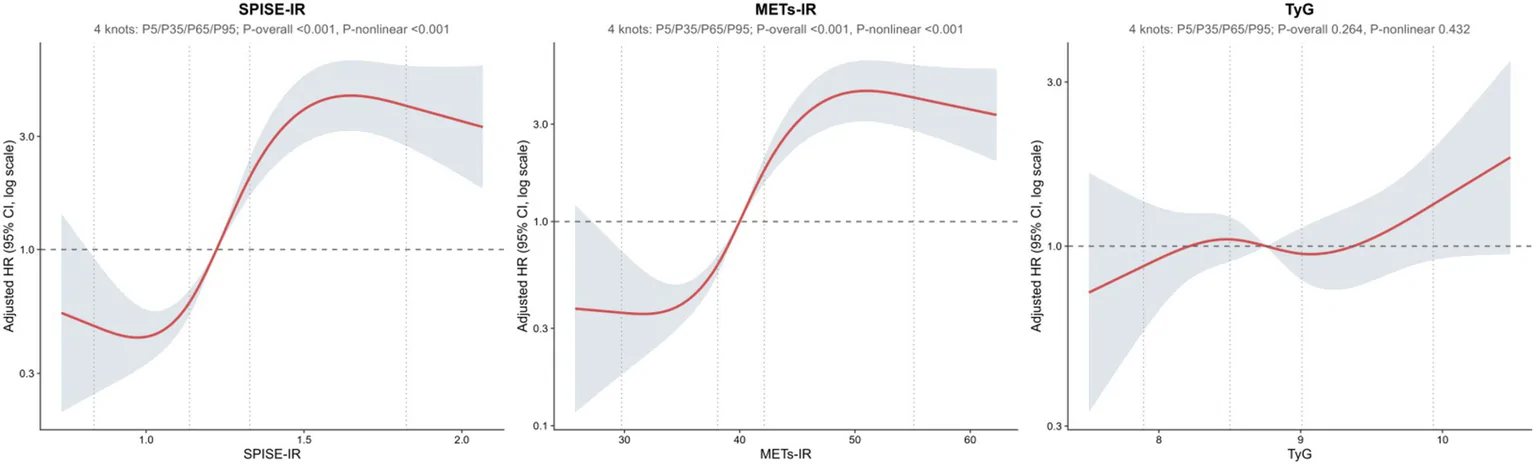
Fully adjusted RCS associations of SPISE-IR, METs-IR, and TyG with 18-month MACE risk. Red lines represent adjusted HRs and shaded areas represent 95% CIs. The horizontal dashed line indicates HR = 1, and vertical dotted lines indicate the four knots. Median index values were used as references; knot locations and *p* values are reported in Table 11.

### 3.5 Predictive performance and component-level comparisons

SPISE-IR and METs-IR showed good predictive performance for MACE (Figure 4A; Table 11), with AUCs of 0.79 (95% CI: 0.746–0.833) and 0.801 (95% CI: 0.760–0.843), respectively, with no significant difference between them (*p* = 0.226). TyG showed poor discrimination (AUC = 0.539, 95% CI: 0.486–0.593).

**Figure 4:**
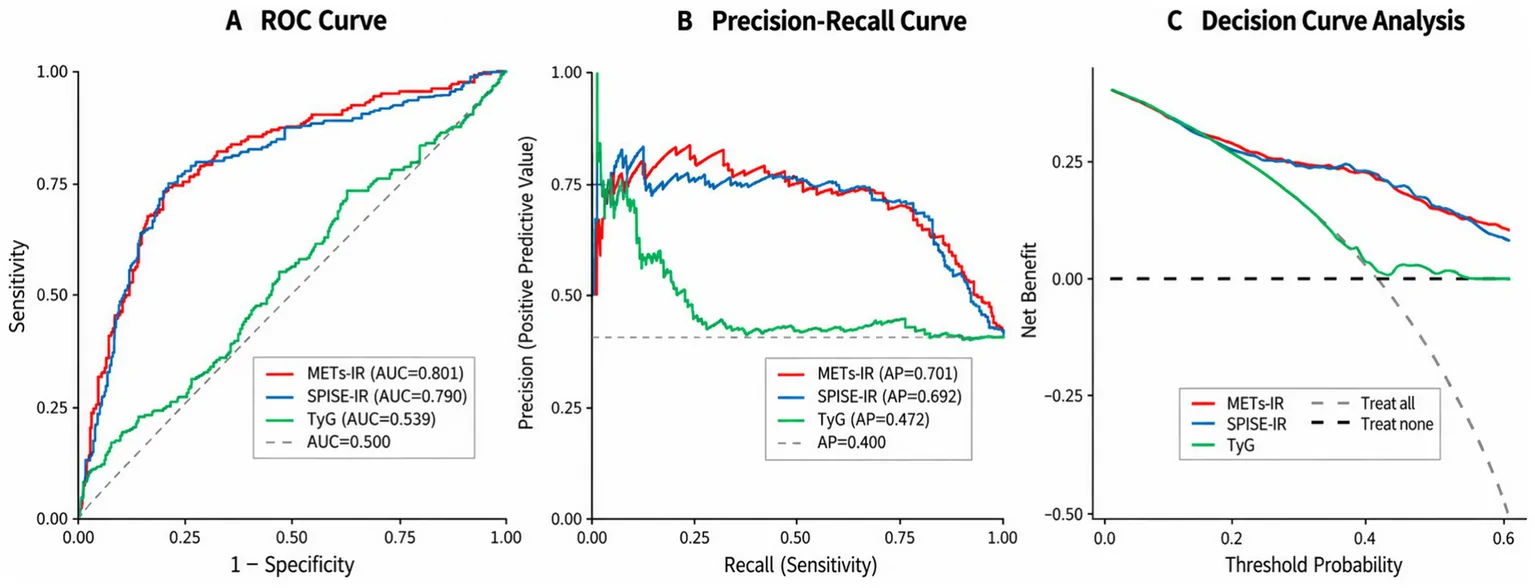
Diagnostic performance evaluation of METS-IR, SPISE-IR and TyG index. **(A)** Receiver operating characteristic (ROC) curves of three indicators for predicting the outcome, with corresponding AUC values and 95% confidence intervals displayed in the legend. The diagonal dashed line represents the reference line with no diagnostic value. **(B)** Precision-recall (PR) curves for each index, and the PR-AUC of each curve is marked in the legend; the diagonal dashed line indicates the baseline random classification level. **(C)** Decision curve analysis (DCA) of the three predictive models. The horizontal yellow line represents the net benefit of the “Treat None” strategy, the red line represents the “Treat All” strategy, and the green, blue and purple curves correspond to the net clinical benefit of prediction models based on METS-IR, SPISE-IR and TyG, respectively, across different threshold probabilities.

PR curve (Figure 4B) analysis showed higher precision–recall performance for SPISE-IR (AUC-PR = 0.688) and METs-IR (AUC-PR = 0.697) compared with TyG (AUC-PR = 0.47). DCA (Figure 4C) demonstrated higher net clinical benefit for SPISE-IR and METs-IR across a wide range of threshold probabilities (10–60%).

In component-level comparisons, SPISE-IR and METs-IR achieved 18-month AUCs of 0.787 and 0.800, respectively, outperforming their individual components but showing similar performance to the corresponding combined component models (AUCs: 0.790 and 0.792; Tables 12, 13 and Figures 5, 6). TyG had an AUC of 0.536, compared with 0.591 for the triglyceride-plus-fasting-glucose model (Table 14; Figure 7). After addition to the baseline clinical model, SPISE-IR and METs-IR did not differ significantly from their component models in AUC, NRI, or IDI. In contrast, TyG performed significantly worse than its component model (ΔAUC = −0.016, *p* = 0.032; NRI = −0.282, *p* = 0.048; IDI = −0.017, *p* = 0.012; Table 15). Minor differences from the conventional AUCs in Table 11 reflect the use of an 18-month time-dependent IPCW estimator.

**Table 12:** Precision–recall curve performance metrics.

Index	AP	95% CI for AP	PR-AUC	95% CI for PR-AUC	Best-F1 cut-off	Precision	Recall	F1
SPISE-IR	0.692	0.614–0.769	0.688	0.610–0.768	1.237	0.667	0.796	0.726
METs-IR	0.701	0.630–0.774	0.696	0.625–0.772	41.013	0.698	0.738	0.718
TyG	0.472	0.409–0.543	0.470	0.405–0.542	7.471	0.402	0.995	0.572

AP, average precision; CI, confidence interval; PR-AUC, area under the precision–recall curve; F1, F1 score; SPISE-IR, single-point insulin sensitivity estimator–insulin resistance index; METS-IR, metabolic score for insulin resistance; TyG, triglyceride–glucose index. The best-F1 cut-off represents the threshold yielding the maximum F1 score. Higher AP and PR-AUC values indicate better discriminative performance.

**Table 13:** Apparent net benefit at prespecified decision thresholds.

Model	Threshold probability	Net benefit	Standardized net benefit	TP	FP	FN	TN
SPISE-IR	0.20	0.258	0.645	175	207	16	79
SPISE-IR	0.30	0.220	0.550	160	128	31	158
SPISE-IR	0.40	0.212	0.529	141	60	50	226
METs-IR	0.20	0.271	0.678	180	202	11	84
METs-IR	0.30	0.228	0.568	166	134	25	152
METs-IR	0.40	0.210	0.525	139	58	52	228
TyG	0.20	0.251	0.626	191	286	0	0
TyG	0.30	0.143	0.358	191	286	0	0
TyG	0.40	0.019	0.047	93	126	98	160

DCA, decision curve analysis; TP, true positive; FP, false positive; FN, false negative; TN, true negative; SPISE-IR, single-point insulin sensitivity estimator–insulin resistance index; METS-IR, metabolic score for insulin resistance; TyG, triglyceride–glucose index.

**Figure 5:**
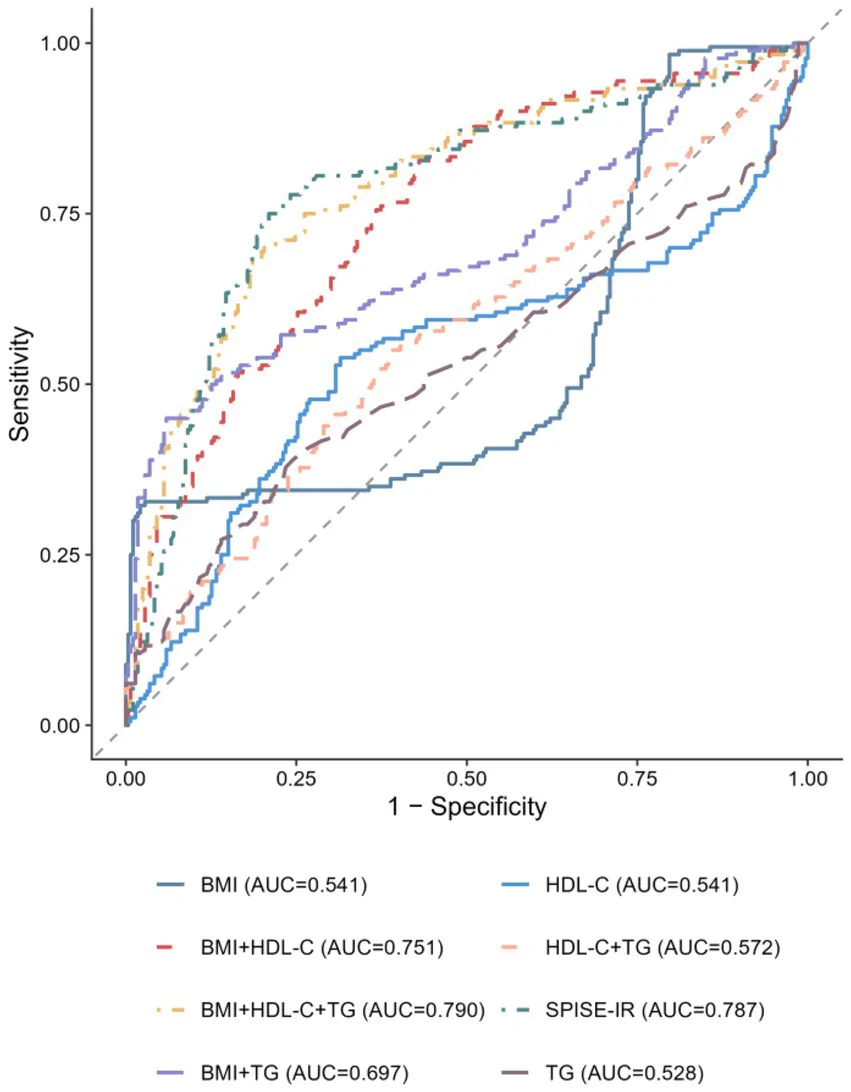
Eighteen-month time-dependent ROC curves for SPISE-IR and its component models. Dynamic AUCs were estimated using inverse-probability-of-censoring weighting. The diagonal dashed line represents chance-level discrimination.

**Figure 6:**
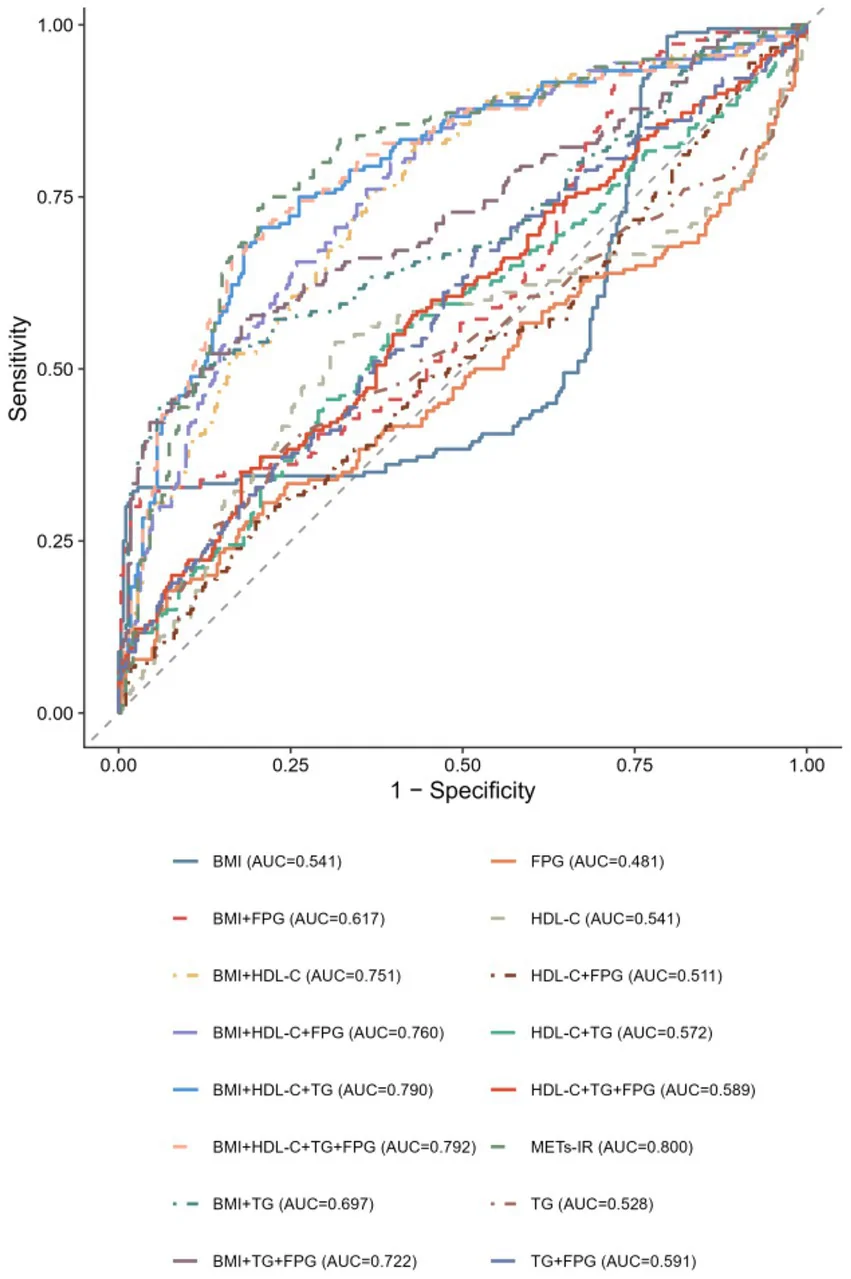
Eighteen-month time-dependent ROC curves for METs-IR and its component models. Dynamic AUCs were estimated using inverse-probability-of-censoring weighting. The diagonal dashed line represents chance-level discrimination.

**Table 14:** Model 2, Model 3, and Model 4 compared to Model 1 in terms of NRI and IDI.

Compare	Metric	Estimate and 95%CI	*p*-value
Model 2 (METS-IR) vs. Model 1	NRI	0.9975 (0.8379 to 1.1570)	<0.001
	IDI	0.1838 (0.1491 to 0.2185)	<0.001
Model 3 (SPISE-IR) vs. Model 1	NRI	1.1023 (0.9484 to 1.2562)	<0.001
	IDI	0.1952 (0.1581 to 0.2323)	<0.001
Model 4 (TyG) vs. Model 1	NRI	0.1242 (−0.0583 to 0.3066)	0.182
	IDI	0.0063 (−0.0023 to 0.0148)	0.149

NRI, net reclassification improvement; IDI, integrated discrimination improvement; METs-IR, metabolic insulin resistance index; SPISE-IR, simple proxy insulin resistance index; TyG, triglyceride-glucose index; 95% CI, 95% confidence interval. Continuous (category-free) NRI was used, and no risk-category thresholds were applied. The 95% confidence intervals for NRI and IDI were calculated from asymptotic standard errors using the normal approximation.

**Figure 7:**
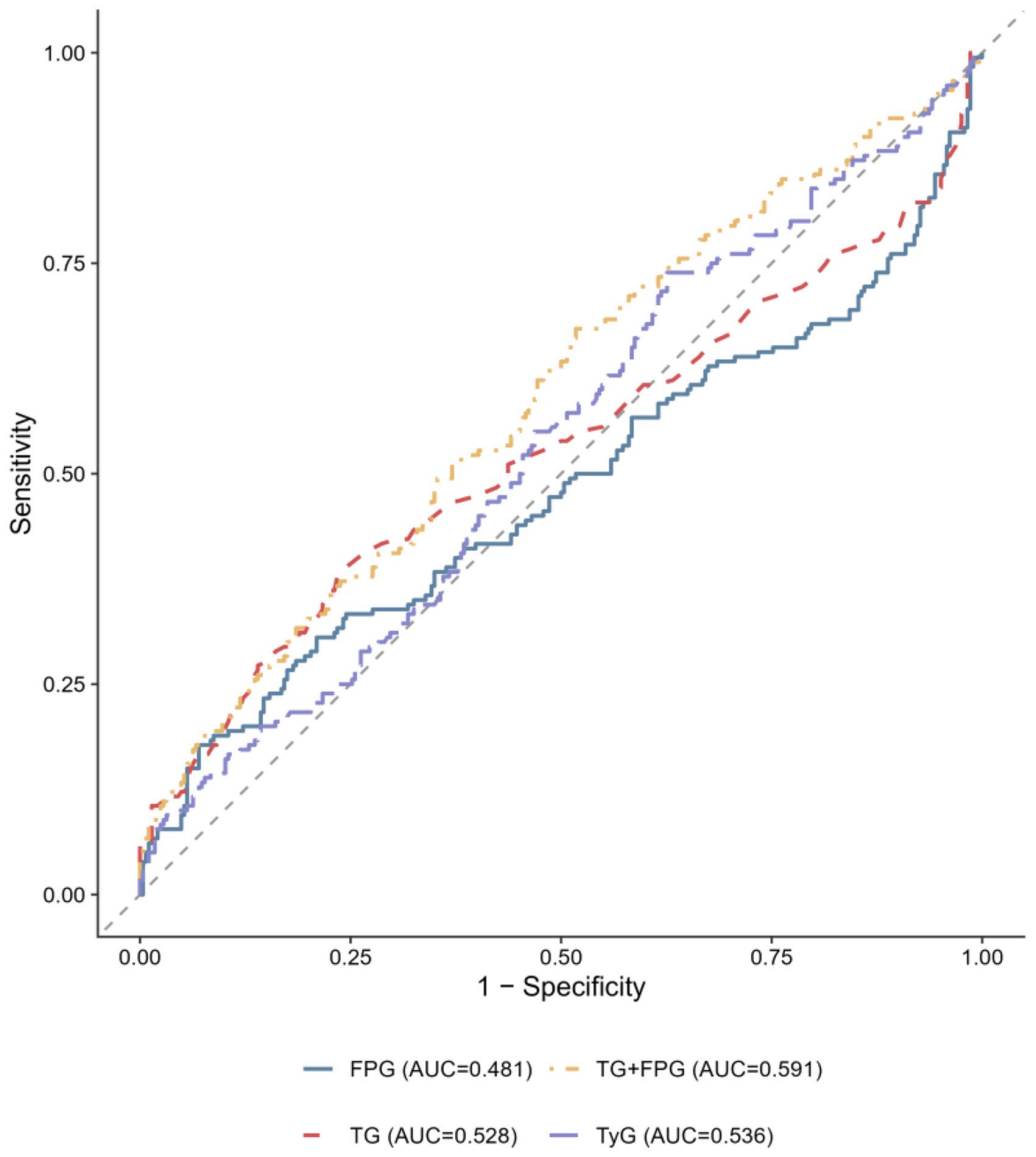
Eighteen-month time-dependent ROC curves for TyG and its component models. Dynamic AUCs were estimated using inverse-probability-of-censoring weighting. The diagonal dashed line represents chance-level discrimination.

**Table 15:** Eighteen-month time-dependent AUCs for SPISE-IR and its component models.

Model	18-month AUC (95% CI)
SPISE-IR	0.787 (0.742–0.831)
BMI	0.541 (0.483–0.598)
HDL-C	0.541 (0.483–0.599)
Triglycerides	0.528 (0.471–0.586)
BMI + HDL-C	0.751 (0.706–0.797)
HDL-C + triglycerides	0.572 (0.518–0.627)
BMI + triglycerides	0.697 (0.645–0.749)
BMI + HDL-C + triglycerides	0.790 (0.746–0.833)

AUC, area under curve; 95% CI, 95% confidence interval.

### 3.6 Survival analysis

KM survival curves demonstrated a clear stepwise reduction in MACE-free survival across increasing SPISE-IR and METs-IR quartiles (Figures 8A,B). Patients in the highest quartiles of SPISE-IR and METs-IR showed significantly earlier event occurrence and lower event-free survival probability compared with the lowest quartiles (log-rank *p* < 0.001 for both). The separation of survival curves was evident early during follow-up and persisted throughout the 18-month observation period. In contrast, no significant differences in survival distribution were observed across TyG quartiles (Figure 8C), with overlapping survival curves throughout follow-up.

**Figure 8:**
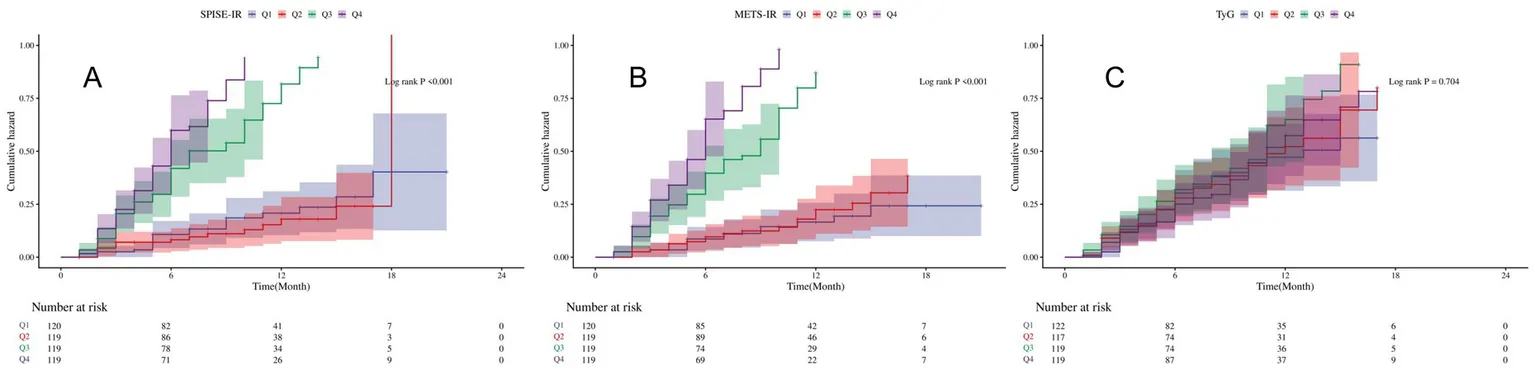
Cumulative hazard curves stratified by quartiles of SPISE-IR, METS-IR and TyG index. **(A)** Cumulative hazard curves for four quartile groups (Q1–Q4) of SPISE-IR. Log-rank test *p* < 0.001 indicated significant inter-group differences in cumulative event risk. **(B)** Cumulative hazard curves across quartiles of METS-IR, with Log-rank *p* < 0.001 demonstrating statistically distinct cumulative hazard among subgroups. **(C)** Cumulative hazard curves stratified by TyG quartiles; Log-rank *p* = 0.704 showed no significant difference in event risk between quartile groups. Shaded bands represent 95% confidence intervals for each curve. The tables at the bottom of each subplot display the number of participants remaining at risk at each time point (months). Q1 = lowest quartile, Q4 = highest quartile of each insulin resistance index.

### 3.7 Incremental predictive performance

The addition of SPISE-IR or METs-IR to the baseline clinical model significantly improved predictive performance, as demonstrated by both reclassification and discrimination metrics (Table 16). SPISE-IR provided the greatest improvement in NRI and IDI compared with the baseline model, indicating superior incremental value in risk stratification. METs-IR also significantly enhanced model performance, although to a slightly lesser extent. In contrast, incorporation of TyG did not result in meaningful improvement in model performance.

**Table 16:** ROC curve table for SPISE-IR, METs-IR, and TyG in predicting MACE.

Variable	AUC	95% CI	*p*-value	Cutoff	Sensitivity	Specificity	Youden index
METS-IR	0.801	0.760–0.843	<0.001	41.206	0.728	0.797	0.525
SPISE-IR	0.790	0.746–0.833	<0.001	1.273	0.743	0.790	0.534
TyG	0.539	0.486–0.593	0.144	8.461	0.733	0.374	0.107

ROC, receiver operating characteristic; AUC, area under curve; MACE, major adverse cardiovascular events; SPISE-IR, simple proxy insulin resistance index; METs-IR, metabolic insulin resistance index; TyG, triglyceride-glucose index; 95% CI, 95% confidence interval.

### 3.8 Subgroup consistency analysis

Stratified Cox regression analyses using the Model 3 covariate set showed that SPISE-IR was significantly associated with MACE in all prespecified subgroups, with no evidence of interaction (all *p* for interaction >0.05; Figure 9). For METs-IR, significant interactions were observed for age (*p* for interactio*n* = 0.010), sex (0.034), smoking status (0.004), and hypertension (0.009), whereas interactions for chronic kidney disease (0.714), prior myocardial infarction (0.163), and HF subtype (0.100) were not significant (Figure 10). TyG was not associated with MACE overall; an association was observed only among smokers (HR = 1.71, 95% CI: 1.05–2.76, *p* = 0.030), and the interaction across HF subtypes was significant (*p* for interactio*n* = 0.045; Figure 11).

**Figure 9:**
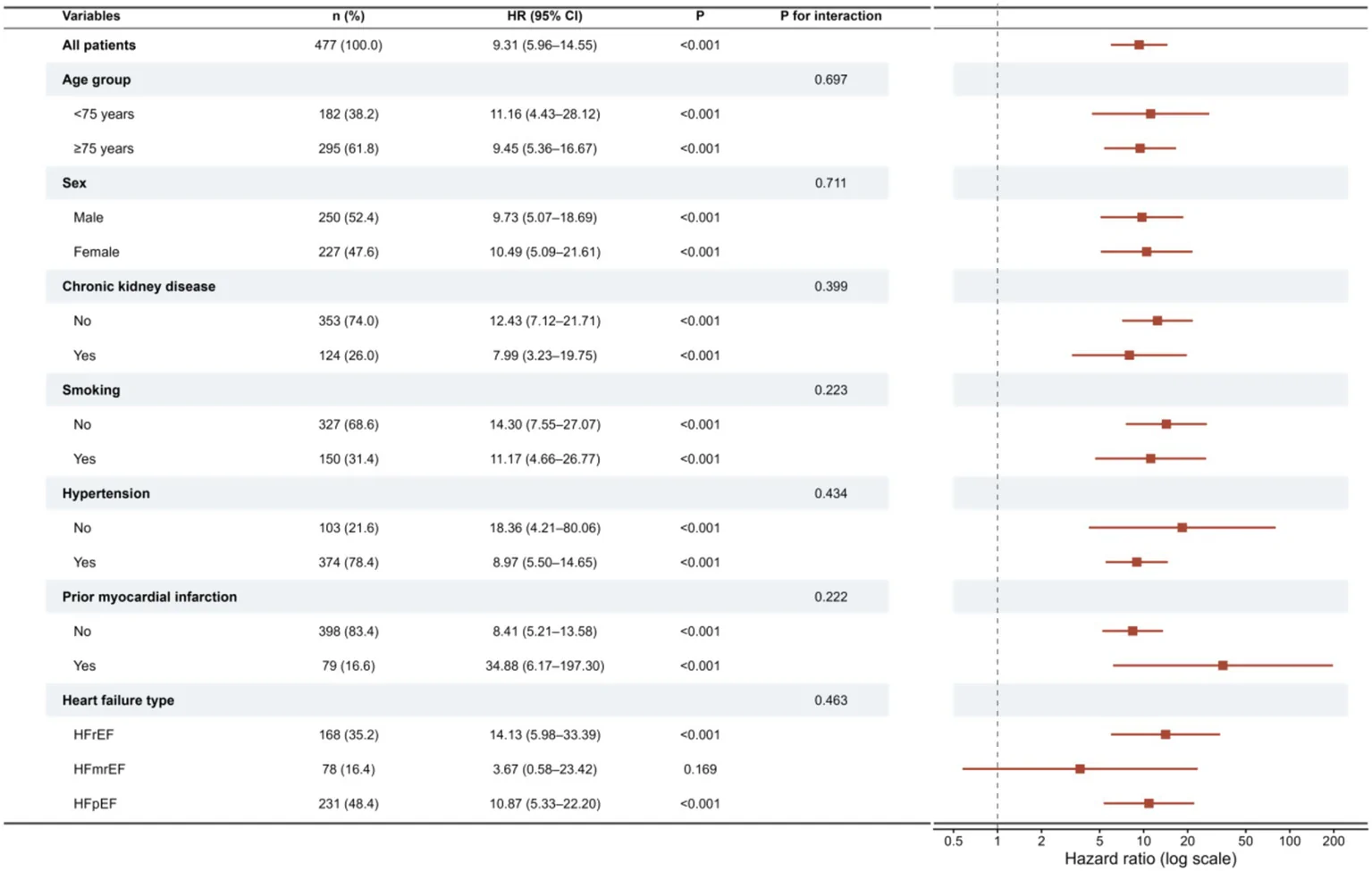
According to the subgroup analysis results of SPISE-IR. Adjusted hazard ratios (HR) and 95% confidence intervals (CIs) were calculated across clinical subgroups including age, gender, smoking status, hypertension, chronic kidney disease (CKD), heart failure subtypes (HFmrEF, HFpEF, HFrEF), and myocardial infarction (MI) history. The column of *p* for interaction was used to test the heterogeneity of association effects among different subgroups. The vertical dashed line at HR = 1 represents the null effect reference; solid dots denote point estimates of HR, and horizontal lines represent corresponding 95% CIs.

**Figure 10:**
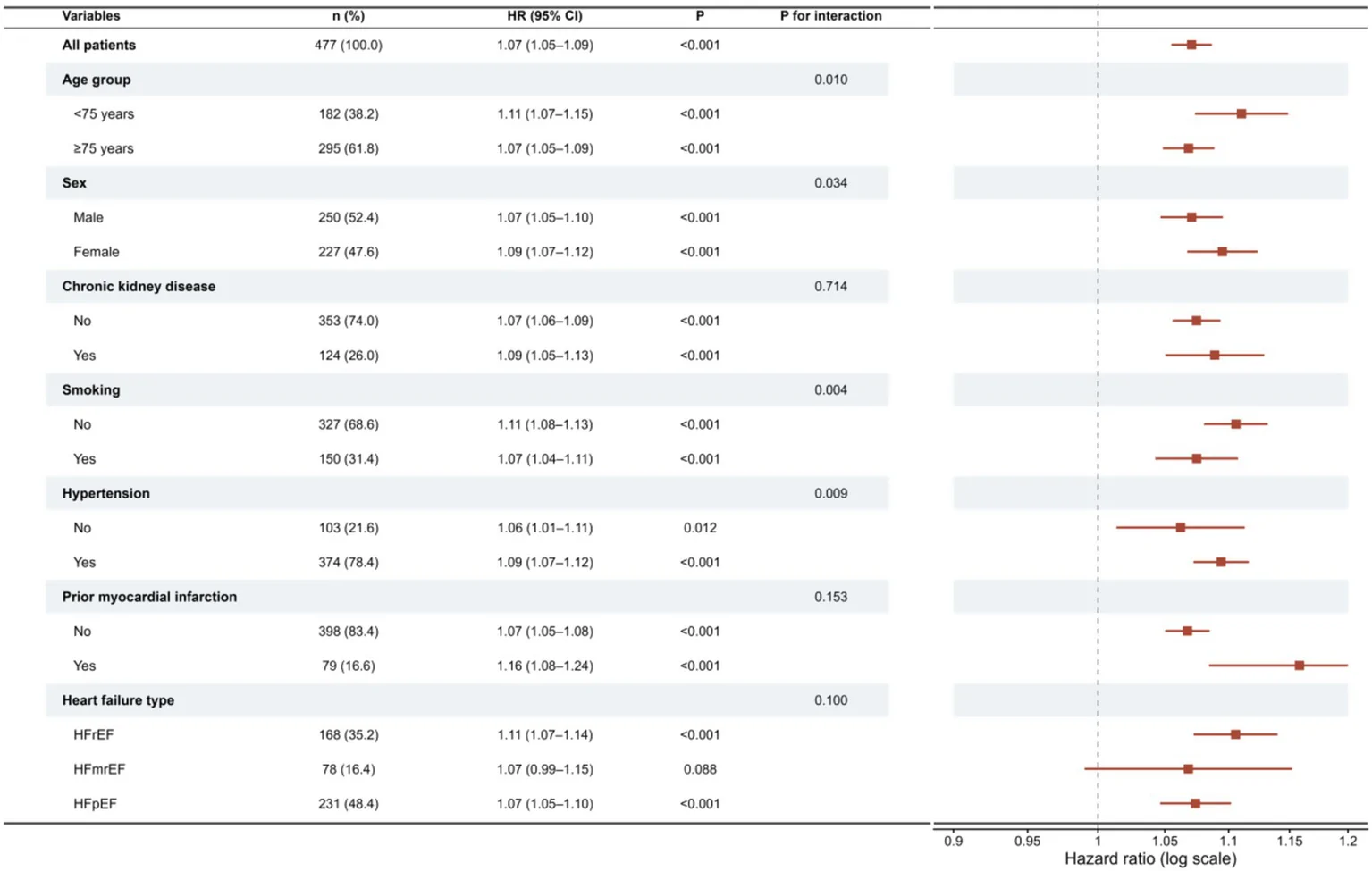
According to the subgroup analysis results of METs-IR. All hazard ratios (HR) and corresponding 95% confidence intervals (CI) were adjusted for confounders. Stratification factors included age, gender, smoking status, hypertension, chronic kidney disease (CKD), heart failure phenotype (HFmrEF/HFpEF/HFrEF), and myocardial infarction (MI) history. The *p* for interaction column tests whether the association effect differs significantly between subgroups. The vertical dashed line at HR = 1 represents the null association reference; solid black dots indicate point estimates of HR, and horizontal lines represent 95% CIs.

**Figure 11:**
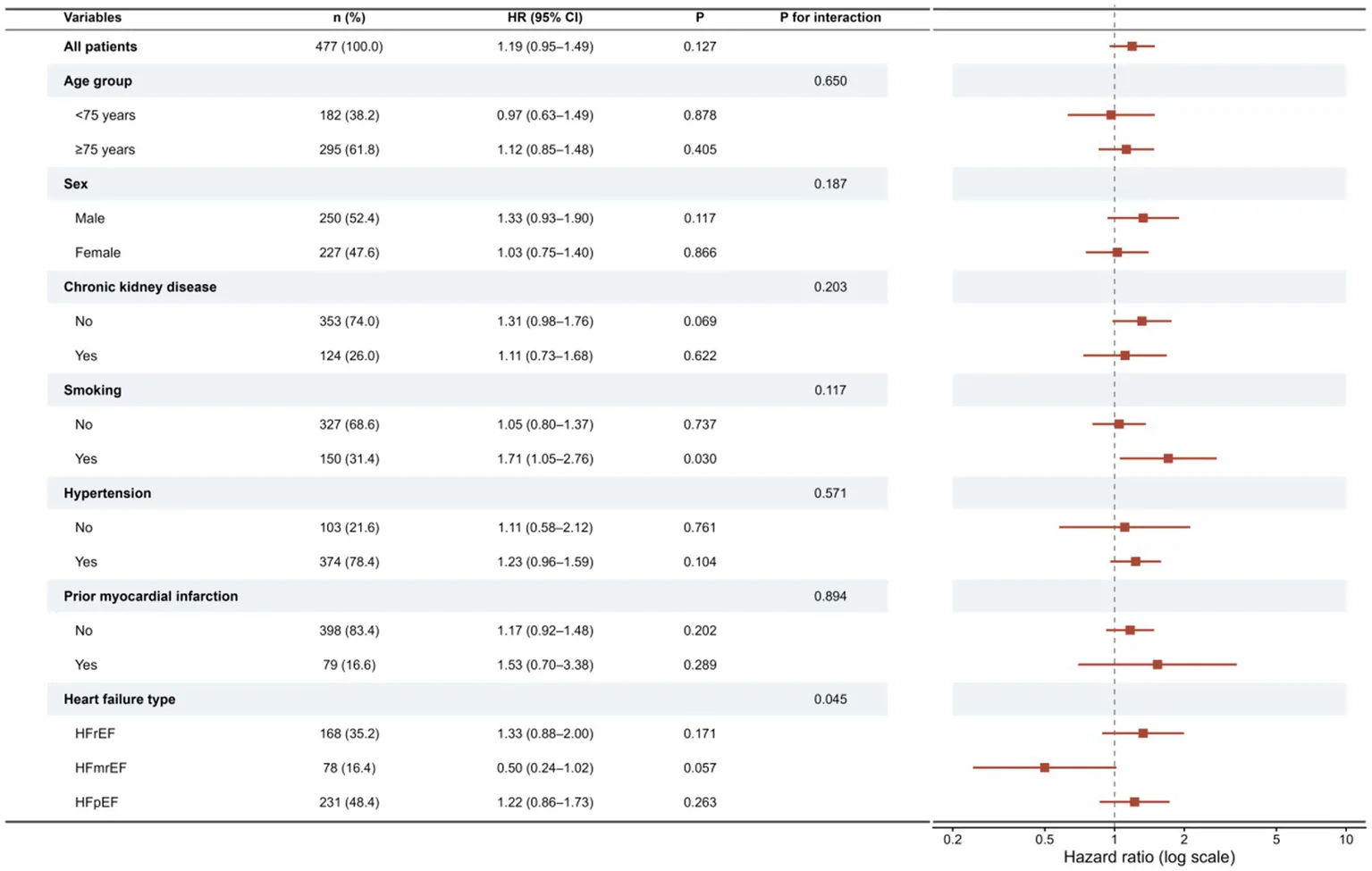
According to the subgroup analysis results of TyG. The adjusted hazard ratios (HR) with corresponding 95% confidence intervals (CI) were calculated across predefined clinical subgroups, including age, gender, smoking status, hypertension, chronic kidney disease (CKD), heart failure subtypes (HFmrEF/HFpEF/HFrEF), and history of myocardial infarction (MI). The *p* for interaction values were presented to test the heterogeneity of effect estimates across subgroups. The vertical dashed line at HR = 1 represents the null effect threshold; horizontal lines denote 95% CIs, and solid dots stand for point HR estimates.

### 3.9 Internal and center-based external validation

Bootstrap validation modestly reduced model performance: optimism-corrected AUCs were 0.705 for the baseline model, 0.806 for clinical+METs-IR, 0.818 for clinical+SPISE-IR, and 0.706 for clinical+TyG, with calibration slopes of 0.733, 0.746, 0.818, and 0.727, respectively (Table 17; Figure 12).

**Table 17:** Definition and event count of the strict MACE endpoint.

Item	Number
Total participants	477
Primary MACE events	191
Recurrent-angina events excluded	24
MACE subtype missing and censored	1
Strict MACE events (nonfatal MI, malignant arrhythmia, heart failure rehospitalization, or cardiovascular death)	166

Strict MACE excluded recurrent angina. The patient with a missing MACE subtype was censored at the recorded event time.

**Figure 12:**
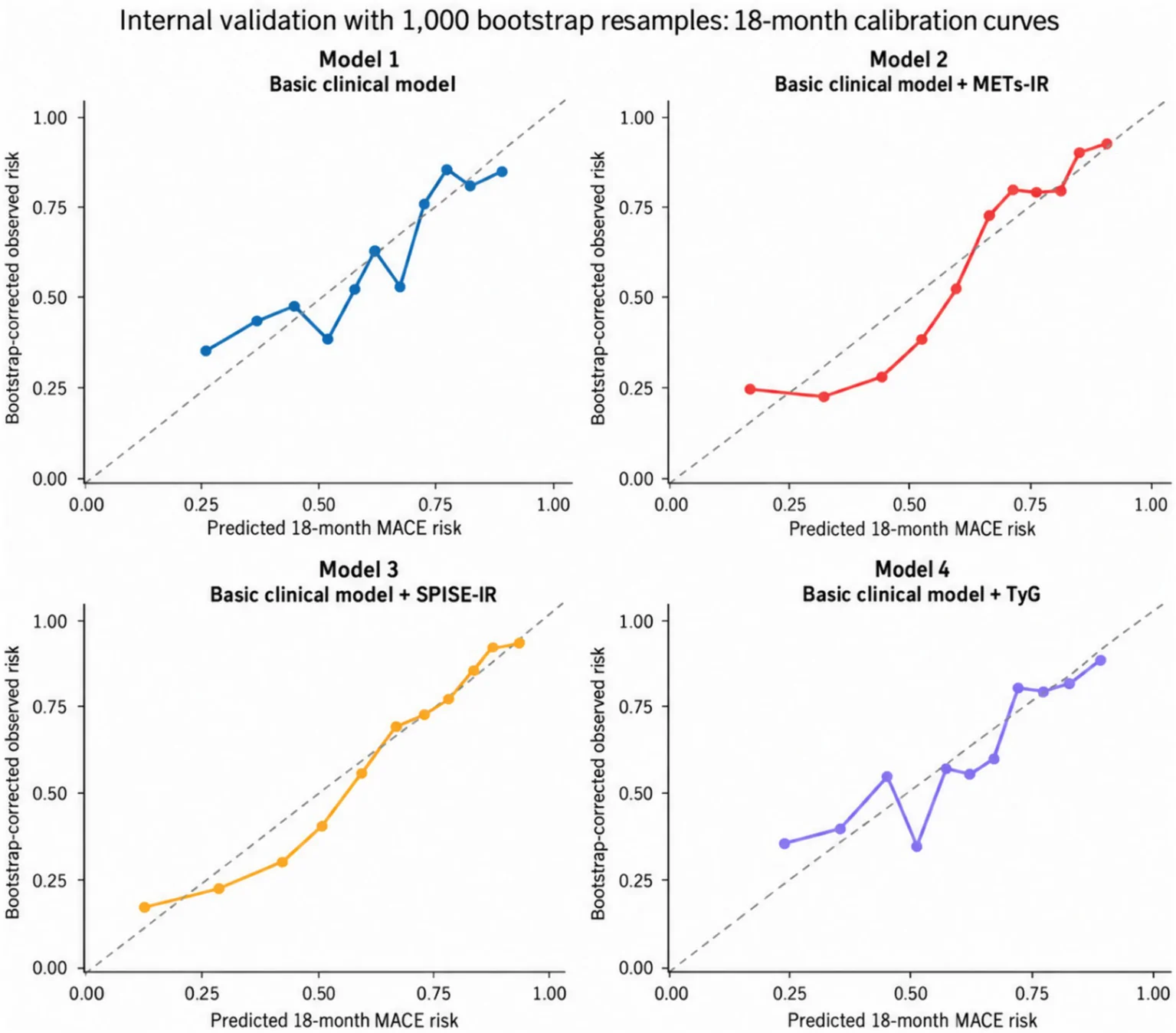
Optimism-corrected 18-month calibration curves from 1,000 bootstrap resamples. All four prediction models completed 1,000 resamples. Variability at high predicted risks reflects the smaller number of participants in this range and model complexity.

In external validation, the corresponding AUC/Brier score/calibration slope values were 0.649/0.245/0.472, 0.806/0.185/0.718, 0.804/0.188/0.741, and 0.665/0.242/0.482 (Tables 18–20; Figure 13). METs-IR and SPISE-IR improved AUC by 0.157 and 0.155, respectively, with lower Brier scores and positive NRI and IDI estimates. TyG provided limited improvement (ΔAUC = 0.016), while DCA confirmed greater net benefit for the METs-IR and SPISE-IR models (Table 21; Figure 14).

**Table 18:** Comparison of fully adjusted associations for primary and strict MACE endpoints.

Index	Endpoint	Events, *n*	Adjusted HR (95% CI)	*p* value
SPISE-IR	Primary endpoint: all MACE	191	9.31 (5.96–14.55)	<0.001
METs-IR	Primary endpoint: all MACE	191	1.07 (1.05–1.09)	<0.001
SPISE-IR	Strict endpoint: recurrent angina excluded	166	10.83 (6.71–17.45)	<0.001
METs-IR	Strict endpoint: recurrent angina excluded	166	1.07 (1.06–1.09)	<0.001

Adjusted estimates are from Model 3. Strict MACE excluded recurrent angina; the patient with a missing MACE subtype was censored at the event time.

**Table 19:** Cox regression results for the strict MACE endpoint.

Index	Model	*N*	Strict MACE events	HR (95% CI)	*p* value
SPISE-IR	Model 1: unadjusted	477	166	7.75 (5.15–11.67)	<0.001
SPISE-IR	Model 2: age and sex	477	166	10.02 (6.51–15.41)	<0.001
SPISE-IR	Model 3: fully adjusted	477	166	10.83 (6.71–17.45)	<0.001
METs-IR	Model 1: unadjusted	477	166	1.07 (1.06–1.08)	<0.001
METs-IR	Model 2: age and sex	477	166	1.08 (1.06–1.09)	<0.001
METs-IR	Model 3: fully adjusted	477	166	1.07 (1.06–1.09)	<0.001

Model 1 was unadjusted. Model 2 was adjusted for age and sex. Model 3 was adjusted for age, sex, smoking, hypertension, chronic kidney disease, NYHA functional class, prior myocardial infarction, LVEF, LDH, albumin, eGFR, CK-MB, NT-proBNP, cardiac troponin I, red blood cell count, platelet count, absolute neutrophil count, and C-reactive protein. SPISE-IR and METs-IR were evaluated in separate models.

**Table 20:** Bootstrap internal validation of the four clinical prediction models.

Model	Apparent AUC	Optimism	Corrected AUC	Apparent slope	Corrected slope	Resamples
Baseline clinical	0.745	0.040	0.705	1.000	0.733	1000
Clinical + METs-IR	0.837	0.031	0.806	1.000	0.746	1000
Clinical + SPISE-IR	0.846	0.029	0.818	1.000	0.818	1000
Clinical + TyG	0.748	0.042	0.706	1.000	0.727	1000

Validation Model 1 was the baseline clinical prediction model; Model 2 added METs-IR; Model 3 added SPISE-IR; and Model 4 added TyG. These labels are distinct from the Cox adjustment Models 1–3 in Table 2. Optimism was estimated using 1,000 bootstrap resamples.

**Figure 13:**
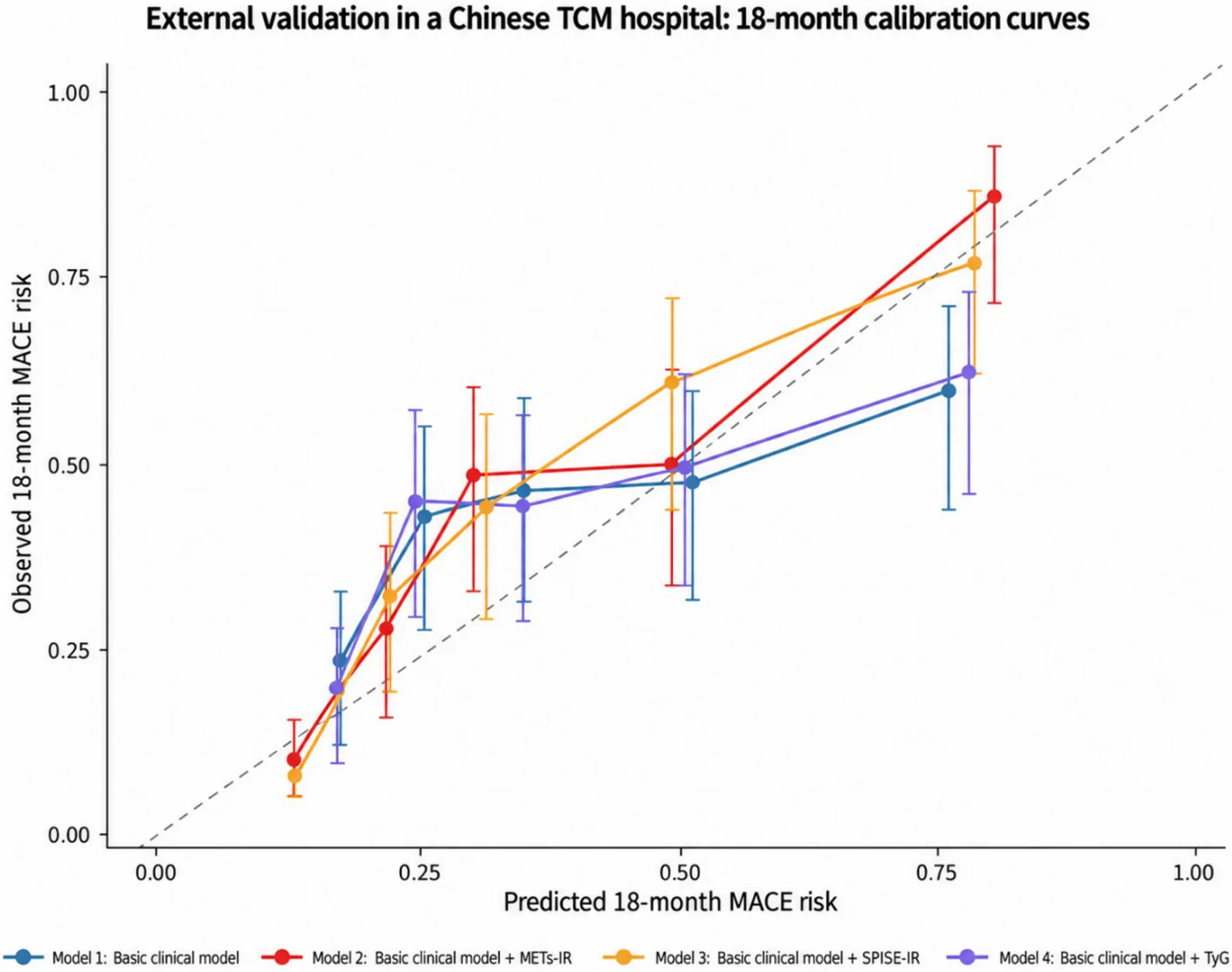
Eighteen-month calibration curves in the center-based external validation cohort. Points show quintile-specific Kaplan–Meier observed risks, with 95% CIs. Model coefficients and the 18-month baseline survival estimated in the derivation cohort were fixed and were not refitted.

**Table 21:** Fixed modeling rules for center-based external validation.

Item	Fixed rule
Derivation center	Zhejiang Xinhua Hospital
External validation center	Zhejiang Provincial Hospital of Traditional Chinese Medicine
Admissions excluded from derivation center	2018 and 2019
Prediction horizon	18 months
Categorical-variable coding	Sex, smoking, hypertension, CKD, and prior MI coded as 0/1; NYHA entered categorically
Continuous-variable handling	Original units; linear terms; no centering, standardization, or log transformation
Missing-data handling	No missing values in analysis variables; complete-case analysis
Risk threshold	Continuous predicted risks; no data-driven cutoff

**Figure 14:**
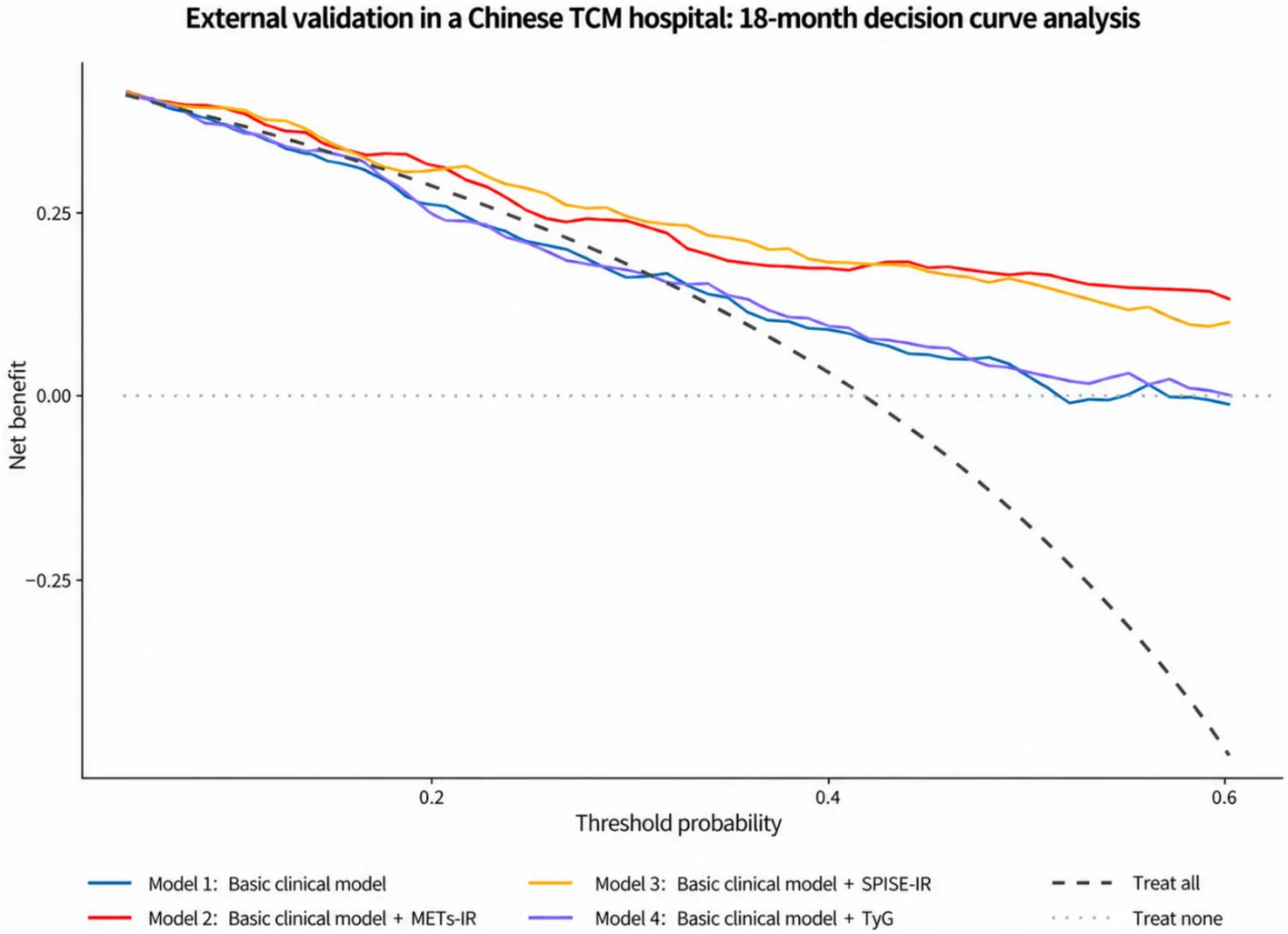
Eighteen-month decision curves in the center-based external validation cohort. Time-to-event outcomes were corrected using inverse-probability-of-censoring weighting; higher net benefit indicates greater potential clinical utility.

### 3.10 Center and enrollment-period sensitivity analyses

When both hospitals were restricted to the shared 2020–2024 enrollment window, center-by-index interaction was significant for SPISE-IR (*p* for interactio*n* = 0.016), but not for METs-IR (*p* = 0.219) or TyG (*p* = 0.711; Tables 22, 23 and Figure 15).

**Table 22:** Eighteen-month baseline survival estimates from the derivation models.

Model	Time, months	Centering	Baseline cumulative hazard	Baseline survival
Baseline clinical	18	Derivation-sample mean	0.09280	0.91138
Clinical + METs-IR	18	Derivation-sample mean	0.13426	0.87436
Clinical + SPISE-IR	18	Derivation-sample mean	0.13727	0.87173
Clinical + TyG	18	Derivation-sample mean	0.09306	0.91114

Model coefficients and baseline survival estimates were obtained only from the Zhejiang Xinhua Hospital derivation cohort and were fixed during evaluation in the validation cohort.

**Table 23:** Eighteen-month performance in the center-based external validation cohort.

Model	Derivation *N*/events	Validation *N*/events	AUC (95% CI)	Brier score (95% CI)	Calibration slope	Resamples
Baseline clinical	210/71	223/93	0.649 (0.573–0.723)	0.245 (0.213–0.279)	0.472	1000
Clinical + METs-IR	210/71	223/93	0.806 (0.752–0.858)	0.185 (0.157–0.215)	0.718	1000
Clinical + SPISE-IR	210/71	223/93	0.804 (0.743–0.856)	0.188 (0.158–0.218)	0.741	1000
Clinical + TyG	210/71	223/93	0.665 (0.597–0.736)	0.242 (0.211–0.273)	0.482	1000

**Figure 15:**
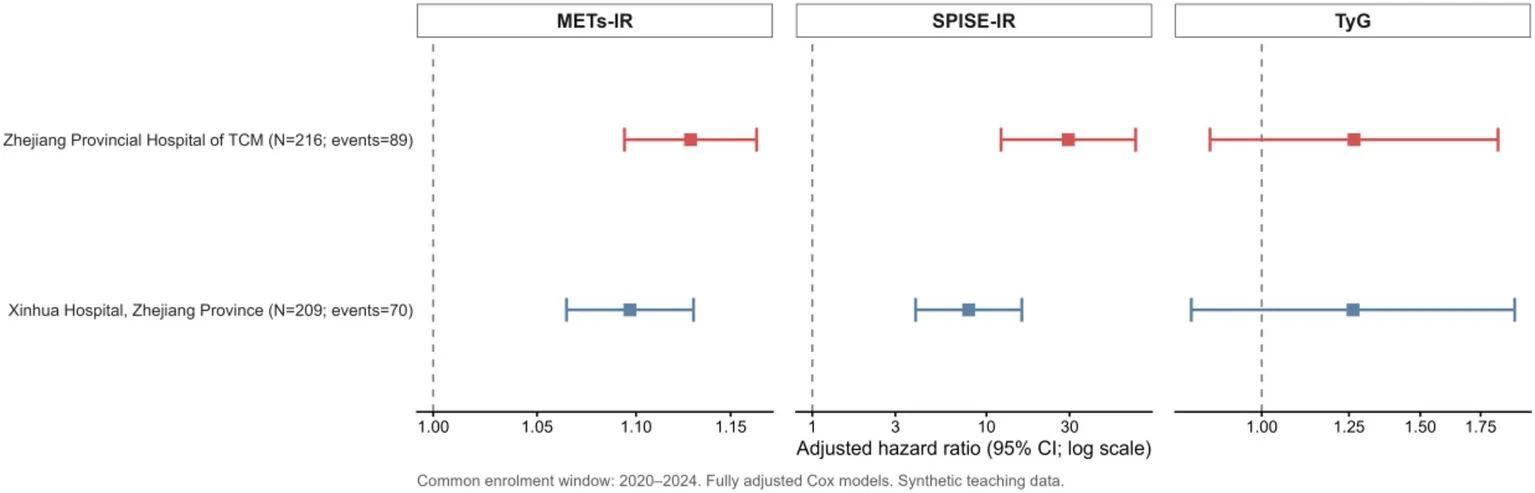
Center-specific sensitivity analyses under calendar-aligned enrollment windows. Fully adjusted HRs and 95% CIs are shown for METs-IR, SPISE-IR, and TyG by center. The panel restricts both hospitals to the shared 2020–2024 enrollment window. Models used the Model 3 covariate set.

In the full center-specific analysis, SPISE-IR remained positively associated with MACE at Zhejiang Provincial Hospital of Traditional Chinese Medicine (*n* = 223; 93 events; HR = 29.33, 95% CI 12.29–70.00; *p* < 0.001) and Zhejiang Xinhua Hospital (*n* = 254; 98 events; HR = 6.04, 95% CI 3.33–10.96; *p* < 0.001), although effect magnitude differed (*p* for interactio*n* = 0.003; Tables 24, 25). The consistent direction, but differing magnitude, warrants cautious interpretation of a single pooled estimate.

**Table 24:** Incremental external-validation performance after addition of each insulin resistance index.

Comparison	Delta AUC (95% CI)	Delta Brier (95% CI)	NRI (95% CI)	IDI (95% CI)
Clinical + METs-IR vs. baseline	0.157 (0.111–0.204)	−0.060 (−0.078 to −0.042)	1.035 (0.796–1.248)	0.169 (0.127–0.210)
Clinical + SPISE-IR vs. baseline	0.155 (0.112–0.199)	−0.057 (−0.075 to −0.039)	0.955 (0.721–1.169)	0.152 (0.116–0.188)
Clinical + TyG vs. baseline	0.016 (0.000–0.032)	−0.004 (−0.010–0.002)	0.205 (−0.064–0.452)	0.016 (0.005–0.028)

Delta values compare each index-augmented model with the baseline clinical model; Delta Brier <0 indicates lower prediction error. Confidence intervals were obtained from 1,000 paired bootstrap resamples of the validation cohort, without re-estimating model coefficients. Continuous NRI was used.

**Table 25:** Center-by-index interaction tests in the shared enrollment window.

Enrollment window	Index	*p* for center interaction
Both centers restricted to 2020–2024	SPISE-IR	0.016
Both centers restricted to 2020–2024	METs-IR	0.219
Both centers restricted to 2020–2024	TyG	0.711

Models used the Model 3 covariate set and additionally included center and the corresponding center-by-index interaction. *p* for interaction <0.05 indicates between-center heterogeneity.

## 4 Discussion

This study included a multicenter retrospective cohort of 477 patients with HF and T2DM, and evaluated the association and predictive value of three insulin resistance surrogate indices (SPISE-IR, METs-IR, and TyG) for short-term MACE within 18 months. Both SPISE-IR and METs-IR were independently associated with MACE and showed stable predictive performance, whereas TyG showed no significant association or predictive ability. RCS analyses demonstrated significant nonlinear associations for SPISE-IR and METs-IR, whereas no significant overall or nonlinear association was observed for TyG; subgroup analyses showed a consistent SPISE-IR association but heterogeneity in the METs-IR association across several clinical strata (Table 26).

**Table 26:** Center-specific fully adjusted associations between SPISE-IR and MACE.

Center	*N*	MACE events	HR	95% CI	*p* value	*p* for interaction
Zhejiang Provincial Hospital of Traditional Chinese Medicine	223	93	29.33	12.29–70.00	<0.001	0.003
Zhejiang Xinhua Hospital	254	98	6.04	3.33–10.96	<0.001	0.003

HR, hazard ratio; CI, confidence interval; MACE, major adverse cardiovascular events.

Center-specific Cox models used the Model 3 covariate set.

Heart failure rehospitalization accounted for nearly three quarters of MACE, indicating that the composite endpoint predominantly reflected recurrent decompensation. Nevertheless, SPISE-IR and METs-IR remained associated with both heart-failure-rehospitalization and non-heart-failure-rehospitalization MACE, and their associations persisted after recurrent angina was excluded. Mechanistically, insulin resistance may impair myocardial substrate utilization and mechano-energetic efficiency and contribute to subclinical ventricular dysfunction and reduced functional reserve, thereby increasing susceptibility to congestion and recurrent decompensation (20, 21). These findings support the robustness of the primary results, although the limited numbers of myocardial infarctions, malignant arrhythmias, and cardiovascular deaths precluded reliable analyses of individual hard endpoints.

In patients with HF and type 2 diabetes, IR reflects not only metabolic disturbance but also a condition closely linked to disease progression and prognosis. Clinically, these patients often show rapidly declining exercise capacity, unstable volume status, and recurrent worsening of symptoms even during relatively stable phases, which cannot be fully explained by glucose or lipid levels alone. At the myocardial level, impaired insulin signaling reduces glucose uptake and shifts energy utilization toward fatty acid oxidation. Although this shift maintains energy supply to some extent, it increases oxygen consumption and reduces metabolic efficiency, leading to mitochondrial stress, increased ROS production, and reduced energetic reserve. Over time, this limits the heart’s ability to respond to hemodynamic stress (22, 23).

Structural remodeling appears alongside these metabolic changes. Abnormal insulin-related signaling promotes activation of pathways such as mTOR and MAPK, leading to cardiomyocyte hypertrophy and fibroblast activation (24). With disease progression, increased collagen deposition and extracellular matrix remodeling contribute to reduced myocardial compliance and impaired diastolic function (25, 26). These changes are not always reflected by a reduced ejection fraction but are closely associated with dyspnea, reduced exercise tolerance, and volume-related symptoms in clinical practice. Electrical instability and inflammation further contribute to adverse outcomes. Changes in ion channel function and action potential properties increase susceptibility to arrhythmias (27, 28). At the same time, activation of NF-κB and NLRP3-related inflammatory pathways enhances cytokine release, which aggravates endothelial dysfunction and microvascular perfusion abnormalities (29–31). Under conditions such as acute stress or fluid overload, this underlying state may more easily trigger clinical events.

SPISE-IR and METs-IR incorporate BMI and HDL-C in addition to conventional lipid and glucose parameters, allowing a more comprehensive reflection of metabolic abnormalities. METs-IR further integrates fasting glucose, triglycerides, HDL-C, and BMI, providing a broader assessment of systemic IR burden (32). In contrast, TyG only reflects the relationship between fasting glucose and triglycerides and does not account for adiposity or protective lipid factors, which may limit its usefulness in patients with multiple comorbidities. Previous studies have shown that SPISE-IR predicts long-term cardiovascular outcomes in metabolic syndrome and diabetes (33), while METs-IR is associated with adverse outcomes in heart failure (34). Our findings extend their value to short-term MACE and show that both indices remain predictive beyond traditional markers such as NT-proBNP and LVEF, suggesting that metabolic risk and structural cardiac dysfunction reflect different aspects of disease biology.

Component-level analyses showed that SPISE-IR and METs-IR outperformed each individual metabolic variable but provided predictive performance comparable to models combining all their components. Thus, their principal advantage is not additional prognostic information beyond BMI, HDL-C, triglycerides, and fasting plasma glucose, but the ability to integrate these variables into a single, readily calculated measure. This parsimonious representation may simplify risk stratification, reduce model complexity and multicollinearity, and facilitate clinical application when constructing a multivariable model is impractical. TyG performed significantly worse than the triglyceride-plus-fasting-glucose model, suggesting that its fixed transformation may lose useful component-level information.

The lack of association between TyG and outcomes may be explained by both patient characteristics and biological limitations. The present cohort included older patients with a high burden of comorbidities such as chronic kidney disease and advanced heart failure, which may reduce the ability of TyG to reflect true IR (35–37). In addition, short-term MACE is more strongly influenced by acute inflammation, hemodynamic instability, and neurohormonal activation, whereas TyG mainly reflects long-term metabolic exposure (38), which may reduce its sensitivity in short-term risk prediction. The lower HbA1c observed in patients with MACE may reflect stress-related glucose variability or more intensive treatment in severely ill patients (39), further weakening the association between long-term glycemic measures and short-term outcomes. In this context, SPISE-IR and METs-IR may better capture the combined effects of lipid metabolism, adiposity, and inflammatory status.

In predictive analyses, SPISE-IR and METs-IR showed better discrimination and clinical utility than TyG in ROC, PR, and decision curve analyses across a wide range of thresholds. Adding either SPISE-IR or METs-IR significantly improved the performance of the baseline model, whereas TyG provided no additional value. Subgroup analyses demonstrated no significant effect modification for SPISE-IR. In contrast, the association of METs-IR with MACE varied by age, sex, smoking status, and hypertension. This consistency supports the concept that insulin resistance acts as a common upstream mechanism across different heart failure phenotypes (40). In contrast, TyG remained non-significant overall and across most subgroups, although an isolated association was observed among smokers and interaction by HF subtype was detected.

Bootstrap correction produced only modest reductions in discrimination, and the approximately 0.80 AUCs for the SPISE-IR and METs-IR models were retained in the held-out center, supporting the stability of their predictive value. However, all corrected and external calibration slopes were below 1, indicating residual overfitting and imperfect calibration across centers. Thus, the validation results strengthen the evidence for discrimination and clinical utility but do not eliminate the need for recalibration and further independent validation.

Several limitations should be considered, including the retrospective design, limited follow-up duration, and lack of dynamic metabolic measurements and lifestyle data. Medication exposure was available only in broad categories (antidiabetic and antihypertensive agents); detailed information on individual medication classes, including sodium-glucose cotransporter 2 (SGLT2) inhibitors, beta-blockers, angiotensin-converting enzyme inhibitors, angiotensin receptor blockers, angiotensin receptor-neprilysin inhibitors, mineralocorticoid receptor antagonists, and diuretics, was unavailable from the original medical records. Consequently, individual guideline-directed heart failure therapies and SGLT2 inhibitor use could not be incorporated into the multivariable adjustment or sensitivity analyses. Residual confounding due to between-patient differences in pharmacological treatment therefore cannot be excluded. Despite these limitations, the present study provides consistent evidence supporting the prognostic value of SPISE-IR and METs-IR in this population. In addition, requiring ascertainable 18-month outcomes may have introduced selection bias by excluding patients with incomplete follow-up, although early cardiovascular deaths were retained. Prospective studies with systematic follow-up are warranted. The external validation cohort was drawn from the second hospital within the same regional study and was not a fully independent prospective cohort; therefore, generalizability to other populations and healthcare settings remains uncertain. Although center adjustment and calendar alignment reduced these concerns, residual confounding from unmeasured center- and time-dependent practice changes cannot be excluded.

In conclusion, SPISE-IR and METs-IR better reflect systemic metabolic and inflammatory burden in patients with HF and type 2 diabetes and are reliable predictors of short-term MACE. In contrast, TyG has limited value in this specific clinical setting. Further prospective studies are needed to confirm these findings and to explore whether risk stratification based on IR can guide personalized treatment strategies and improve short-term outcomes.

## Data Availability

The original contributions presented in the study are included in the article/Supplementary material, further inquiries can be directed to the corresponding author.

## References

[1] Palandurkar G Kumar S. Current status of dapagliflozin in congestive heart failure. Cureus. (2022) 14:e29413. doi: 10.7759/cureus.29413, 36304362 PMC9586194

[2] Khan SA Shields S Abusamaan MS Mathioudakis N. Association between dysglycemia and the Charlson comorbidity index among hospitalized patients with diabetes. J Diabetes Complicat. (2022) 36:108305. doi: 10.1016/j.jdiacomp.2022.108305, 36108545 PMC13281902

[3] Chen X Li W Zheng J Huang M Wang J Wu M. Type 2 diabetes mediated heart failure: focus on early recognition and clinical strategies. Front Endocrinol (Lausanne). (2025) 16:1630686. doi: 10.3389/fendo.2025.1630686 PMC12457163 41001676

[4] Mohammadian K Basirian N Fakhar F Keramat S Stanek A. Insulin resistance and platelet hyperactivity: hematological insights and nutritional strategies for vascular protection. Nutrients. (2026) 18:763. doi: 10.3390/nu18050763, 41829931 PMC12987119

[5] Wood N Straw S Scalabrin M Roberts LD Witte KK Bowen TS. Skeletal muscle atrophy in heart failure with diabetes: from molecular mechanisms to clinical evidence. ESC Heart Fail. (2021) 8:3–15. doi: 10.1002/ehf2.13121, 33225593 PMC7835554

[6] Obaid HA Marques-Vidal P. Trends in diabetes mellitus and related costs among hospital admissions in Switzerland, 2012–2020. Diabetol Metab Syndr. (2012) 17:40. doi: 10.1186/s13098-025-01604-z, 39891293 PMC11783757

[7] Angelini G Albanese M Ursi R Lisi F Bellino MC Amato L . Eligibility of outpatients with chronic heart failure for sodium-glucose co-transporter-2 inhibitors. ESC Heart Fail. (2021) 8:2951–8. doi: 10.1002/ehf2.13380, 33991178 PMC8318395

[8] Frampton JE. Empagliflozin: a review in symptomatic chronic heart failure. Drugs. (2022) 82:1591–602. doi: 10.1007/s40265-022-01778-0, 36374374

[9] Tofte N Theilade S Winther SA Birkelund S Goetze JP Hansen TW . Comparison of natriuretic peptides as risk markers for all-cause mortality and cardiovascular and renal complications in individuals with type 1 diabetes. Diabetes Care. (2021) 44:595–603. doi: 10.2337/dc20-2107, 33323477

[10] Tang XF Li QX Han YL Wang X-Z Song Y Zhang Z . Implications of baseline glycemic control by plasma glycated hemoglobin A1c on adverse outcomes in patients with coronary heart disease and type 2 diabetes mellitus: results from the promise study. Heliyon. (2024) 10:e39748. doi: 10.1016/j.heliyon.2024.e39748 PMC11585765 39584103

[11] Abe T Yokota T Fukushima A Kakutani N Katayama T Shirakawa R . Type 2 diabetes is an independent predictor of lowered peak aerobic capacity in heart failure patients with non-reduced or reduced left ventricular ejection fraction. Cardiovasc Diabetol. (2020) 19:142. doi: 10.1186/s12933-020-01114-4, 32950064 PMC7502205

[12] Ma RCW Tam CHT Hou Y Lau ESH Ozaki R Lui JNM . Nt-probnp improves prediction of cardiorenal complications in type 2 diabetes: the Hong Kong diabetes biobank. Diabetologia. (2025) 68:342–56. doi: 10.1007/s00125-024-06299-x, 39508878 PMC11732878

[13] Obrador De Hevia J López-González ÁA Ramírez-Manent JI . Association between alcohol consumption, other healthy habits and sociodemographic variables and the values of different insulin resistance risk scales in 139,634 Spanish workers. Healthcare (Basel). (2025) 13:921. doi: 10.3390/healthcare13080921, 40281870 PMC12026883

[14] Stein R Koutny F Riedel J Dörr N Meyer K Colombo M . Single point insulin sensitivity estimator (Spise) as a prognostic marker for emerging dysglycemia in children with overweight or obesity. Meta. (2023) 13:100. doi: 10.3390/metabo13010100, 36677025 PMC9867183

[15] Seo MW Cho W Kim JY. The single point insulin sensitivity estimator (Spise) index as a predictor of metabolic syndrome in Korean adults. Obes Res Clin Pract. (2023) 17:198–202. doi: 10.1016/j.orcp.2023.05.001, 37246046

[16] Alogaili MH Alsallami AA. The Association of the Metabolic Score for insulin resistance (Mets-Ir) with prehypertension in Normoglycemic individuals. Int J Endocrinol Metab. (2024) 22:e145894. doi: 10.5812/ijem-145894, 40071249 PMC11892513

[17] Hasanato R Alshehri NA Binsalamh LF Ismail SM. Triglyceride-glucose index and triglyceride-glucose index-related markers for identifying metabolic syndrome in Saudi Arabia. J Clin Lipidol. (2026) 20:665–70. doi: 10.1016/j.jacl.2026.01.002, 41617626

[18] Guo W Zhao L Mo F Peng C Li L Xu Y . The prognostic value of the triglyceride glucose index in patients with chronic heart failure and type 2 diabetes: a retrospective cohort study. Diabetes Res Clin Pract. (2021) 177:108786. doi: 10.1016/j.diabres.2021.108786, 33812901

[19] Ormazabal V Nair S Elfeky O Aguayo C Salomon C Zuñiga FA. Association between insulin resistance and the development of cardiovascular disease. Cardiovasc Diabetol. (2018) 17:122. doi: 10.1186/s12933-018-0762-4, 30170598 PMC6119242

[20] Gudenkauf B Shaya G Mukherjee M Michos ED Madrazo J Mathews L . Insulin resistance is associated with subclinical myocardial dysfunction and reduced functional capacity in heart failure with preserved ejection fraction. J Cardiol. (2024) 83:100–4. doi: 10.1016/j.jjcc.2023.06.008, 37364818 PMC12973288

[21] Cefalo CMA Riccio A Fiorentino TV Succurro E Perticone M Sciacqua A . Impaired insulin sensitivity measured by estimated glucose disposal rate is associated with decreased myocardial mechano-energetic efficiency in non-diabetic individuals. Eur J Intern Med. (2024) 130:144–50. doi: 10.1016/j.ejim.2024.09.008, 39289108

[22] Zhang L Keung W Samokhvalov V . Role of fatty acid uptake and fatty acid beta-oxidation in mediating insulin resistance in heart and skeletal muscle. Biochim Biophys Acta. (2010) 1801:1–22. doi: 10.1016/j.bbalip.2009.12.006 19782765

[23] Westermeier F Navarro-Marquez M López-Crisosto C Bravo-Sagua R Quiroga C Bustamante M . Defective insulin signaling and mitochondrial dynamics in diabetic cardiomyopathy. Biochim Biophys Acta. (2015) 1853:1113–8. doi: 10.1016/j.bbamcr.2015.02.005, 25686534 PMC4626247

[24] Tao J Yu XL Yuan YJ Shen X Liu J Gu P-P . Dmrt2 interacts with Fxr and improves insulin resistance in adipocytes and a mouse model. Front Endocrinol (Lausanne). (2021) 12:723623. doi: 10.3389/fendo.2021.723623, 35250844 PMC8891600

[25] Fan Y Yan Z Li T Li A Fan X Qi Z . Primordial drivers of diabetes heart disease: comprehensive insights into insulin resistance. Diabetes Metab J. (2024) 48:19–36. doi: 10.4093/dmj.2023.0110, 38173376 PMC10850268

[26] Musale V Murdoch CE Banah AK . Limiting extracellular matrix expansion in diet-induced obese mice reduces cardiac insulin resistance and prevents myocardial remodelling. Mol Metab. (2024) 86:101970. doi: 10.1016/j.molmet.2024.101970 PMC11334943 38908792

[27] Jin L Gao F Jiang T Liu B Li C Qin X . Hyper-O-Glcnacylation impairs insulin response against reperfusion-induced myocardial injury and arrhythmias in obesity. Biochem Biophys Res Commun. (2021) 558:126–33. doi: 10.1016/j.bbrc.2021.04.066 33915326

[28] Ogawa W Araki E Ishigaki Y Hirota Y Maegawa H Yamauchi T . New classification and diagnostic criteria for insulin resistance syndrome. Diabetol Int. (2022) 13:337–43. doi: 10.1007/s13340-022-00570-5, 35463863 PMC8980125

[29] Šebeková K Gurecká R Csongová M Koborová I Repiská G Podracká Ľ. Lean insulin-resistant young adults display increased cardiometabolic risk: a retrospective cross-sectional study. Diabetes Res Clin Pract. (2022) 185:109217. doi: 10.1016/j.diabres.2022.109217, 35114297

[30] Mastrototaro L Roden M. Insulin resistance and insulin sensitizing agents. Metabolism. (2021) 125:154892. doi: 10.1016/j.metabol.2021.154892, 34563556

[31] Hanna A Frangogiannis NG. Inflammatory cytokines and chemokines as therapeutic targets in heart failure. Cardiovasc Drugs Ther. (2020) 34:849–63. doi: 10.1007/s10557-020-07071-0, 32902739 PMC7479403

[32] Qin YN Zheng XP. Association of frailty index with congestive heart failure, all-cause and cardiovascular mortality among individuals with type 2 diabetes: a study from National Health and nutrition examination surveys (Nhanes), 1999–2018. Diabetol Metab Syndr. (2023) 15:210. doi: 10.1186/s13098-023-01165-z, 37875981 PMC10594933

[33] Wani K Khattak MNK Saadawy GM Al-Attas OS Alokail MS Al-Daghri NM. Sex-specific cut-offs of single point insulin sensitivity estimator (Spise) in predicting metabolic syndrome in the Arab adolescents. Diagnostics (Basel). (2023) 13:324. doi: 10.3390/diagnostics13020324, 36673133 PMC9858553

[34] Lin Z Wu Y Zhao D Kang S Zhang M Yang P . Cumulative metabolic score for insulin resistance and heart failure risk: a prospective study. Nutr Metab Cardiovasc Dis. (2026) 36:104634. doi: 10.1016/j.numecd.2026.104634 41936493

[35] Kalyani RR Egan JM. Diabetes and altered glucose metabolism with aging. Endocrinol Metab Clin N Am. (2013) 42:333–47. doi: 10.1016/j.ecl.2013.02.010, 23702405 PMC3664017

[36] Sakaguchi M. Aging-induced dysfunction of adipose tissue and skeletal muscle and its link to insulin resistance. J Biochem. (2026) 179:221–30. doi: 10.1093/jb/mvaf081, 41437560

[37] Spoto B Pisano A Zoccali C. Insulin resistance in chronic kidney disease: a systematic review. Am J Physiol Renal Physiol. (2016) 311:F1087–f108. doi: 10.1152/ajprenal.00340.2016 27707707

[38] Tao LC Xu JN Wang TT Hua F Li J-J. Triglyceride-glucose index as a marker in cardiovascular diseases: landscape and limitations. Cardiovasc Diabetol. (2022) 21:68. doi: 10.1186/s12933-022-01511-x PMC9078015 35524263

[39] Daina MD Vesa CM Ghitea TC . Hyperglycemia in critically ill patients: current approaches and management strategies. In Vivo. (2026) 40:583–99. doi: 10.21873/invivo.14223, 41482394 PMC12764272

[40] Kanyal S Das A Bashir AMD . N‑terminal pro-B-type natriuretic peptide (Nt-probnp) as a biomarker in heart failure with preserved ejection fraction (Hfpef) versus heart failure with reduced ejection fraction (Hfref): the way forward in the age of proteomics. Cureus. (2025) 17:e94162. doi: 10.7759/cureus.94162, 41209853 PMC12595257

