# Supplementary material for: Study on the correlation between SPISE-IR, METs-IR, TyG and short-term adverse cardiovascular events in patients with heart failure and diabetes mellitus
Source: Front Nutr. 2026 Oct 16;13:1938459. doi: 10.3389/fnut.2026.1938459 (PMC13719381; doi:10.3389/fnut.2026.1938459)
Supplement: Supplementary file 1 [file Table_1.docx]

**Supplementary Table 1. Baseline characteristics grouped by TyG quartiles**

| **Variables** | **Total (n = 477)** | **Q1 (n = 122)** | **Q2 (n = 117)** | **Q3 (n = 119)** | **Q4 (n = 119)** | **Statistic** | **P** |
| --- | --- | --- | --- | --- | --- | --- | --- |
| WBC, ×10⁹/L | 6.60 (5.10, 8.20) | 5.90 (4.40, 7.70) | 6.30 (5.00, 7.80) | 6.90 (5.40, 8.30) | 7.00 (5.90, 9.45) | Kruskal-Wallis | <0.001 |
| Age(years) | 78.00 (70.00, 85.00) | 79.00 (70.00, 84.00) | 78.00 (72.00, 83.00) | 80.00 (72.00, 86.00) | 76.00 (66.00, 84.00) | Kruskal-Wallis | 0.046 |
| LVEF (%) | 57.00 (49.00, 64.00) | 57.00 (50.00, 63.75) | 59.00 (50.00, 66.00) | 57.00 (51.00, 62.00) | 55.00 (46.50, 62.00) | Kruskal-Wallis | 0.084 |
| LVDD(mm) | 50.00 (46.00, 54.00) | 49.00 (46.00, 54.00) | 50.00 (46.00, 54.00) | 49.50 (46.55, 53.00) | 50.00 (46.00, 54.50) | Kruskal-Wallis | 0.915 |
| HbA1c(%) | 6.90 (6.30, 7.80) | 6.50 (5.90, 7.00) | 6.80 (6.20, 7.40) | 7.10 (6.30, 7.70) | 7.90 (7.00, 8.70) | Kruskal-Wallis | <0.001 |
| FPG(mmol/L) | 6.72 (4.81, 9.58) | 5.48 (4.30, 7.12) | 6.60 (4.77, 8.96) | 7.55 (5.25, 10.66) | 7.84 (5.33, 12.49) | Kruskal-Wallis | <0.001 |
| TC(mmol/L) | 3.79 (3.06, 4.54) | 3.44 (2.79, 4.05) | 3.45 (2.85, 4.31) | 3.89 (3.13, 4.41) | 4.62 (3.66, 5.64) | Kruskal-Wallis | <0.001 |
| LDL-C(mmol/L) | 1.77 (1.32, 2.45) | 1.56 (1.20, 1.96) | 1.63 (1.22, 2.29) | 1.92 (1.44, 2.38) | 2.55 (1.62, 3.08) | Kruskal-Wallis | <0.001 |
| TG(mmol/L) | 1.12 (0.76, 1.89) | 0.73 (0.54, 0.95) | 0.99 (0.72, 1.40) | 1.30 (0.89, 2.01) | 2.49 (1.52, 3.96) | Kruskal-Wallis | <0.001 |
| HDL-C(mmol/L) | 0.97 (0.57, 1.53) | 1.05 (0.66, 1.64) | 1.01 (0.63, 1.52) | 0.98 (0.49, 1.58) | 0.77 (0.50, 1.37) | Kruskal-Wallis | 0.022 |
| Cr(μmol/L) | 90.00 (69.80, 125.00) | 86.10 (65.90, 113.53) | 87.50 (69.10, 121.40) | 99.70 (79.50, 135.05) | 87.50 (69.70, 122.05) | Kruskal-Wallis | 0.009 |
| LDH(U/L) | 197.00 (165.00, 237.00) | 196.00 (166.00, 244.25) | 187.00 (159.00, 230.00) | 201.00 (166.50, 232.00) | 204.00 (181.00, 252.50) | Kruskal-Wallis | 0.047 |
| ALB(g/L) | 34.80 (31.70, 38.10) | 34.00 (31.33, 37.70) | 34.80 (31.70, 38.00) | 34.60 (32.15, 38.00) | 35.40 (31.65, 38.80) | Kruskal-Wallis | 0.392 |
| ALT(U/L) | 18.00 (12.00, 29.00) | 16.50 (12.00, 25.75) | 19.00 (12.00, 30.00) | 17.00 (12.00, 27.00) | 20.00 (12.00, 34.00) | Kruskal-Wallis | 0.163 |
| AST(U/L) | 22.00 (17.00, 31.00) | 22.50 (17.00, 30.75) | 23.00 (19.00, 32.00) | 21.00 (17.00, 29.50) | 22.00 (17.00, 30.50) | Kruskal-Wallis | 0.660 |
| eGFR, (mL·min⁻¹·1.73m⁻²) | 66.20 (47.40, 90.84) | 72.00 (50.33, 98.45) | 69.30 (48.40, 84.10) | 60.90 (44.42, 80.96) | 67.37 (48.34, 95.25) | Kruskal-Wallis | 0.043 |
| CK-MB(U/L) | 12.60 (6.58, 18.20) | 13.90 (6.82, 18.28) | 10.80 (5.70, 15.00) | 11.80 (7.05, 18.75) | 13.90 (6.55, 19.45) | Kruskal-Wallis | 0.026 |
| NT-proBNP(pg/mL) | 2387.00 (1238.00, 5364.00) | 2671.50 (1531.50, 5601.75) | 2385.50 (1107.00, 4987.00) | 2367.00 (1353.50, 4315.00) | 2226.00 (1046.00, 5860.50) | Kruskal-Wallis | 0.556 |
| TnI(μg/L) | 0.02 (0.01, 0.06) | 0.02 (0.01, 0.05) | 0.02 (0.01, 0.04) | 0.03 (0.01, 0.06) | 0.02 (0.01, 0.10) | Kruskal-Wallis | 0.102 |
| RBC, ×10¹²/L | 4.03 ± 0.76 | 3.91 ± 0.76 | 4.05 ± 0.76 | 4.05 ± 0.74 | 4.13 ± 0.79 | Welch ANOVA | 0.150 |
| PLT, ×10⁹/L | 175.00 (139.00, 218.00) | 170.50 (126.00, 212.50) | 172.00 (139.00, 204.00) | 160.00 (138.00, 208.50) | 190.00 (156.50, 238.00) | Kruskal-Wallis | 0.004 |
| ANC, ×10⁹/L | 4.40 (3.20, 6.00) | 4.00 (2.80, 5.90) | 4.10 (3.20, 5.40) | 4.60 (3.15, 5.95) | 4.60 (3.85, 6.60) | Kruskal-Wallis | 0.002 |
| NEUT(%) | 69.23 (60.40, 76.90) | 69.35 (59.32, 76.95) | 67.60 (59.72, 75.50) | 69.50 (60.51, 76.55) | 69.80 (61.23, 77.40) | Kruskal-Wallis | 0.799 |
| CRP(mg/L) | 5.00 (1.70, 16.28) | 4.20 (1.52, 17.08) | 5.40 (1.70, 16.83) | 4.54 (1.61, 13.93) | 5.91 (2.04, 16.31) | Kruskal-Wallis | 0.887 |
| Height(cm) | 163.00 (156.00, 168.00) | 162.00 (155.25, 168.00) | 165.00 (158.00, 170.00) | 162.00 (156.00, 168.00) | 161.00 (155.50, 167.75) | Kruskal-Wallis | 0.117 |
| Weight(kg) | 64.70 (58.00, 72.00) | 65.00 (57.25, 71.75) | 65.10 (59.00, 73.00) | 63.00 (58.00, 70.00) | 63.00 (56.00, 73.50) | Kruskal-Wallis | 0.720 |
| BMI | 23.64 (21.89, 25.58) | 24.04 (22.43, 26.29) | 23.50 (21.85, 25.25) | 23.46 (22.14, 25.59) | 23.45 (20.94, 25.23) | Kruskal-Wallis | 0.164 |
| SPISE | 8.18 (7.13, 9.27) | 8.80 (7.69, 10.40) | 8.61 (7.89, 9.62) | 7.81 (6.99, 8.77) | 7.14 (6.32, 8.42) | Kruskal-Wallis | <0.001 |
| SPISE IR | 1.22 (1.08, 1.40) | 1.14 (0.96, 1.30) | 1.16 (1.04, 1.27) | 1.28 (1.14, 1.43) | 1.40 (1.19, 1.58) | Kruskal-Wallis | <0.001 |
| METS IR | 39.99 (36.28, 44.73) | 38.21 (33.43, 42.10) | 38.75 (35.73, 42.14) | 41.27 (38.14, 45.61) | 41.86 (38.66, 47.79) | Kruskal-Wallis | <0.001 |
| TyG | 8.76 (8.34, 9.22) | 8.10 (7.93, 8.25) | 8.55 (8.44, 8.65) | 8.98 (8.85, 9.08) | 9.56 (9.34, 9.87) | Kruskal-Wallis | <0.001 |
| Gender |  |  |  |  |  | χ² test | 0.026 |
| Male, n(%) | 250 (52.4) | 67 (54.9) | 73 (62.4) | 58 (48.7) | 52 (43.7) |  |  |
|  |  |  |  |  |  |  |  |
| Female, n(%) | 227 (47.6) | 55 (45.1) | 44 (37.6) | 61 (51.3) | 67 (56.3) |  |  |
| Smoking |  |  |  |  |  | χ² test | 0.610 |
| No, n(%) | 327 (68.6) | 81 (66.4) | 77 (65.8) | 82 (68.9) | 87 (73.1) |  |  |
|  |  |  |  |  |  |  |  |
| Yes, n(%) | 150 (31.4) | 41 (33.6) | 40 (34.2) | 37 (31.1) | 32 (26.9) |  |  |
| Hypertension |  |  |  |  |  | χ² test | 0.959 |
| No, n(%) | 103 (21.6) | 26 (21.3) | 27 (23.1) | 24 (20.2) | 26 (21.8) |  |  |
|  |  |  |  |  |  |  |  |
| Yes, n(%) | 374 (78.4) | 96 (78.7) | 90 (76.9) | 95 (79.8) | 93 (78.2) |  |  |
| CKD |  |  |  |  |  | χ² test | 0.298 |
| No, n(%) | 353 (74.0) | 98 (80.3) | 86 (73.5) | 85 (71.4) | 84 (70.6) |  |  |
|  |  |  |  |  |  |  |  |
| Yes, n(%) | 124 (26.0) | 24 (19.7) | 31 (26.5) | 34 (28.6) | 35 (29.4) |  |  |
| HF |  |  |  |  |  | χ² test | 0.664 |
| HFrEF, n(%) | 168 (35.2) | 38 (31.1) | 47 (40.2) | 41 (34.5) | 42 (35.3) |  |  |
|  |  |  |  |  |  |  |  |
| HFmrEF, n(%) | 78 (16.4) | 20 (16.4) | 16 (13.7) | 18 (15.1) | 24 (20.2) |  |  |
| HFpEF, n(%) | 231 (48.4) | 64 (52.5) | 54 (46.2) | 60 (50.4) | 53 (44.5) |  |  |
| NYHA |  |  |  |  |  | χ² test | 0.991 |
| NYHA II, n(%) | 129 (27.0) | 33 (27.0) | 34 (29.1) | 32 (26.9) | 30 (25.2) |  |  |
|  |  |  |  |  |  |  |  |
| NYHA III, n(%) | 249 (52.2) | 62 (50.8) | 60 (51.3) | 64 (53.8) | 63 (52.9) |  |  |
| NYHA IV, n(%) | 99 (20.8) | 27 (22.1) | 23 (19.7) | 23 (19.3) | 26 (21.8) |  |  |
| MI |  |  |  |  |  | χ² test | 0.074 |
| No, n(%) | 398 (83.4) | 110 (90.2) | 99 (84.6) | 94 (79.0) | 95 (79.8) |  |  |
|  |  |  |  |  |  |  |  |
| Yes, n(%) | 79 (16.6) | 12 (9.8) | 18 (15.4) | 25 (21.0) | 24 (20.2) |  |  |
| PCI |  |  |  |  |  | χ² test | 0.060 |
| No, n(%) | 347 (72.7) | 95 (77.9) | 92 (78.6) | 81 (68.1) | 79 (66.4) |  |  |
|  |  |  |  |  |  |  |  |
| Yes, n(%) | 130 (27.3) | 27 (22.1) | 25 (21.4) | 38 (31.9) | 40 (33.6) |  |  |
| Diabetes |  |  |  |  |  | χ² test | 0.991 |
| Yes, n(%) | 477 (100.0) | 122 (100.0) | 117 (100.0) | 119 (100.0) | 119 (100.0) |  |  |
| Antidiabetic Agents |  |  |  |  |  | χ² test | 0.366 |
| No, n(%) | 70 (14.7) | 22 (18.0) | 18 (15.4) | 12 (10.1) | 18 (15.1) |  |  |
|  |  |  |  |  |  |  |  |
| Yes, n(%) | 407 (85.3) | 100 (82.0) | 99 (84.6) | 107 (89.9) | 101 (84.9) |  |  |
| Antihypertensive Agents |  |  |  |  |  | χ² test | 0.696 |
| No, n(%) | 120 (25.2) | 31 (25.4) | 29 (24.8) | 26 (21.8) | 34 (28.6) |  |  |
|  |  |  |  |  |  |  |  |
| Yes, n(%) | 357 (74.8) | 91 (74.6) | 88 (75.2) | 93 (78.2) | 85 (71.4) |  |  |
| Hospital |  |  |  |  |  | χ² test | 0.304 |
| Zhejiang Provincial Hospital of Chinese Medicine, n(%) | 223 (46.8) | 48 (39.3) | 57 (48.7) | 59 (49.6) | 59 (49.6) |  |  |
|  |  |  |  |  |  |  |  |
| Zhejiang Xinhua Hospital, n(%) | 254 (53.2) | 74 (60.7) | 60 (51.3) | 60 (50.4) | 60 (50.4) |  |  |
| 18-month MACE |  |  |  |  |  | χ² test | 0.436 |
| Non-MACE, n(%) | 286 (60.0) | 78 (63.9) | 74 (63.2) | 66 (55.5) | 68 (57.1) |  |  |
|  |  |  |  |  |  |  |  |
| With MACE, n(%) | 191 (40.0) | 44 (36.1) | 43 (36.8) | 53 (44.5) | 51 (42.9) |  |  |

TyG, triglyceride-glucose index; MACE, major adverse cardiovascular events; WBC, white blood cell count; LVEF, left ventricular ejection fraction; LVDD, left ventricular end-diastolic diameter; HbA1c, glycated hemoglobin; FPG, fasting plasma glucose; TC, total cholesterol; LDL-C, low-density lipoprotein cholesterol; TG, triglyceride; HDL-C, high-density lipoprotein cholesterol; Cr, serum creatinine; LDH, lactate dehydrogenase; ALB, albumin; ALT, alanine aminotransferase; AST, aspartate aminotransferase; eGFR, estimated glomerular filtration rate; CK-MB, creatine kinase-MB isoenzyme; NT-proBNP, N-terminal pro-B-type natriuretic peptide; TnI, cardiac troponin I; RBC, red blood cell count; PLT, platelet count; ANC, absolute neutrophil count; NEUT, neutrophil percentage; CRP, C-reactive protein; BMI, body mass index; CKD, chronic kidney disease; HF, heart failure; HFrEF, heart failure with reduced ejection fraction; HFmrEF, heart failure with mildly reduced ejection fraction; HFpEF, heart failure with preserved ejection fraction; NYHA, New York Heart Association cardiac function classification; MI, myocardial infarction; PCI, percutaneous coronary intervention.
