# Supplementary material for: Study on the correlation between SPISE-IR, METs-IR, TyG and short-term adverse cardiovascular events in patients with heart failure and diabetes mellitus
Source: Front Nutr. 2026 Oct 16;13:1938459. doi: 10.3389/fnut.2026.1938459 (PMC13719381; doi:10.3389/fnut.2026.1938459)
Supplement: Supplementary file 2 [file Table_2.docx]

**Supplementary Table 2. Baseline characteristics grouped by SPISE-IR quartiles**

| **Variables** | **Total (n = 477)** | **Q1 (n = 120)** | **Q2 (n = 119)** | **Q3 (n = 119)** | **Q4 (n = 119)** | **Statistic** | **P** |
| --- | --- | --- | --- | --- | --- | --- | --- |
| WBC, ×10⁹/L | 6.60 (5.10, 8.20) | 6.65 (5.00, 8.20) | 6.70 (5.10, 8.20) | 6.50 (5.25, 8.55) | 6.40 (5.15, 8.10) | Kruskal-Wallis | 0.835 |
| Age(years) | 78.00 (70.00, 85.00) | 78.50 (73.00, 84.00) | 79.00 (69.00, 84.50) | 77.00 (68.50, 84.00) | 78.00 (71.00, 86.00) | Kruskal-Wallis | 0.526 |
| LVEF (%) | 57.00 (49.00, 64.00) | 58.00 (49.75, 66.00) | 59.00 (50.00, 66.00) | 56.00 (45.00, 61.00) | 57.00 (50.00, 62.00) | Kruskal-Wallis | 0.023 |
| LVDD(mm) | 50.00 (46.00, 54.00) | 49.00 (44.15, 52.00) | 49.00 (45.00, 54.00) | 51.00 (48.00, 55.00) | 50.00 (46.05, 55.00) | Kruskal-Wallis | 0.007 |
| HbA1c(%) | 6.90 (6.30, 7.80) | 7.00 (6.30, 7.80) | 6.90 (6.20, 7.60) | 6.90 (6.30, 7.70) | 6.90 (6.20, 8.20) | Kruskal-Wallis | 0.821 |
| FPG(mmol/L) | 6.72 (4.81, 9.58) | 6.38 (5.05, 9.14) | 7.17 (5.39, 9.91) | 5.70 (3.87, 7.89) | 7.69 (4.92, 11.48) | Kruskal-Wallis | <0.001 |
| TC(mmol/L) | 3.79 (3.06, 4.54) | 3.75 (3.10, 4.36) | 3.95 (2.95, 4.32) | 3.78 (3.05, 4.73) | 3.75 (3.12, 5.02) | Kruskal-Wallis | 0.754 |
| LDL-C(mmol/L) | 1.77 (1.32, 2.45) | 1.71 (1.25, 2.16) | 1.80 (1.34, 2.36) | 1.88 (1.33, 2.66) | 1.85 (1.35, 2.76) | Kruskal-Wallis | 0.103 |
| TG(mmol/L) | 1.12 (0.76, 1.89) | 0.84 (0.62, 1.33) | 0.99 (0.76, 1.25) | 1.45 (1.02, 1.89) | 1.92 (0.77, 2.97) | Kruskal-Wallis | <0.001 |
| HDL-C(mmol/L) | 0.97 (0.57, 1.53) | 1.13 (0.65, 1.66) | 1.08 (0.75, 1.46) | 0.70 (0.49, 1.14) | 0.82 (0.49, 1.81) | Kruskal-Wallis | <0.001 |
| Cr(μmol/L) | 90.00 (69.80, 125.00) | 86.10 (65.80, 113.85) | 84.10 (69.00, 108.50) | 95.00 (75.70, 137.30) | 94.10 (73.95, 133.90) | Kruskal-Wallis | 0.001 |
| LDH(U/L) | 197.00 (165.00, 237.00) | 196.00 (162.00, 253.25) | 198.00 (164.00, 242.50) | 198.00 (164.00, 238.50) | 197.00 (177.00, 230.00) | Kruskal-Wallis | 0.901 |
| ALB(g/L) | 34.80 (31.70, 38.10) | 34.45 (31.40, 37.90) | 34.80 (32.15, 37.90) | 35.30 (32.45, 38.90) | 34.30 (31.05, 37.95) | Kruskal-Wallis | 0.218 |
| ALT(U/L) | 18.00 (12.00, 29.00) | 17.00 (12.75, 29.00) | 19.00 (12.00, 27.00) | 18.00 (12.00, 28.00) | 17.00 (12.00, 32.50) | Kruskal-Wallis | 0.903 |
| AST(U/L) | 22.00 (17.00, 31.00) | 22.00 (19.00, 32.00) | 24.00 (17.00, 33.00) | 21.00 (17.00, 27.50) | 21.00 (17.00, 30.00) | Kruskal-Wallis | 0.388 |
| eGFR, (mL·min⁻¹·1.73m⁻²) | 66.20 (47.40, 90.84) | 72.31 (50.82, 92.56) | 70.90 (51.75, 94.95) | 64.59 (43.44, 87.02) | 59.82 (43.65, 80.84) | Kruskal-Wallis | 0.005 |
| CK-MB(U/L) | 12.60 (6.58, 18.20) | 12.45 (5.90, 18.85) | 11.80 (6.35, 15.70) | 11.60 (7.05, 16.80) | 13.80 (8.14, 19.20) | Kruskal-Wallis | 0.149 |
| NT-proBNP(pg/mL) | 2387.00 (1238.00, 5364.00) | 2532.50 (1017.25, 4885.75) | 2156.00 (1242.50, 3410.00) | 2385.50 (1359.00, 6136.00) | 2824.00 (1289.00, 6748.50) | Kruskal-Wallis | 0.041 |
| TnI(μg/L) | 0.02 (0.01, 0.06) | 0.02 (0.01, 0.08) | 0.02 (0.01, 0.05) | 0.02 (0.01, 0.06) | 0.02 (0.01, 0.05) | Kruskal-Wallis | 0.713 |
| RBC, ×10¹²/L | 4.03 ± 0.76 | 3.95 ± 0.68 | 4.11 ± 0.75 | 4.05 ± 0.82 | 4.02 ± 0.79 | Welch ANOVA | 0.351 |
| PLT, ×10⁹/L | 175.00 (139.00, 218.00) | 167.00 (140.00, 215.25) | 175.00 (139.00, 212.00) | 187.00 (144.50, 227.00) | 167.00 (131.00, 215.50) | Kruskal-Wallis | 0.187 |
| ANC, ×10⁹/L | 4.40 (3.20, 6.00) | 4.35 (3.00, 6.15) | 4.50 (3.30, 5.80) | 4.30 (3.45, 6.25) | 4.50 (3.15, 5.90) | Kruskal-Wallis | 0.914 |
| NEUT(%) | 69.23 (60.40, 76.90) | 71.93 (60.42, 77.53) | 67.40 (59.61, 75.65) | 68.10 (61.00, 78.10) | 69.10 (59.57, 75.15) | Kruskal-Wallis | 0.309 |
| CRP(mg/L) | 5.00 (1.70, 16.28) | 4.71 (1.52, 17.61) | 5.20 (1.40, 15.30) | 5.47 (2.24, 20.09) | 4.29 (1.67, 13.77) | Kruskal-Wallis | 0.332 |
| Height(cm) | 163.00 (156.00, 168.00) | 161.00 (155.00, 168.00) | 165.00 (158.00, 171.00) | 165.00 (156.50, 170.00) | 160.00 (155.50, 167.00) | Kruskal-Wallis | 0.007 |
| Weight(kg) | 64.70 (58.00, 72.00) | 54.50 (49.00, 60.00) | 65.00 (57.50, 70.00) | 67.50 (62.00, 72.00) | 72.00 (65.00, 79.50) | Kruskal-Wallis | <0.001 |
| BMI | 23.64 (21.89, 25.58) | 21.52 (17.19, 23.65) | 24.05 (22.96, 25.10) | 23.45 (22.28, 25.40) | 27.26 (23.14, 35.54) | Kruskal-Wallis | <0.001 |
| SPISE | 8.18 (7.13, 9.27) | 10.41 (9.91, 11.59) | 8.68 (8.43, 8.89) | 7.62 (7.40, 7.92) | 6.24 (5.59, 6.71) | Kruskal-Wallis | <0.001 |
| SPISE IR | 1.22 (1.08, 1.40) | 0.96 (0.86, 1.01) | 1.15 (1.13, 1.19) | 1.31 (1.26, 1.35) | 1.60 (1.49, 1.79) | Kruskal-Wallis | <0.001 |
| METS IR | 39.99 (36.28, 44.73) | 32.74 (30.12, 35.84) | 38.41 (36.74, 39.85) | 41.40 (39.97, 43.17) | 49.51 (45.91, 54.23) | Kruskal-Wallis | <0.001 |
| TyG | 8.76 (8.34, 9.22) | 8.43 (8.12, 8.88) | 8.62 (8.34, 9.02) | 8.81 (8.38, 9.17) | 9.18 (8.78, 9.68) | Kruskal-Wallis | <0.001 |
| Gender |  |  |  |  |  | χ² test | 0.209 |
| Male, n(%) | 250 (52.4) | 59 (49.2) | 67 (56.3) | 69 (58.0) | 55 (46.2) |  |  |
|  |  |  |  |  |  |  |  |
| Female, n(%) | 227 (47.6) | 61 (50.8) | 52 (43.7) | 50 (42.0) | 64 (53.8) |  |  |
| Smoking |  |  |  |  |  | χ² test | 0.941 |
| No, n(%) | 327 (68.6) | 81 (67.5) | 82 (68.9) | 80 (67.2) | 84 (70.6) |  |  |
|  |  |  |  |  |  |  |  |
| Yes, n(%) | 150 (31.4) | 39 (32.5) | 37 (31.1) | 39 (32.8) | 35 (29.4) |  |  |
| Hypertension |  |  |  |  |  | χ² test | 0.084 |
| No, n(%) | 103 (21.6) | 32 (26.7) | 30 (25.2) | 24 (20.2) | 17 (14.3) |  |  |
|  |  |  |  |  |  |  |  |
| Yes, n(%) | 374 (78.4) | 88 (73.3) | 89 (74.8) | 95 (79.8) | 102 (85.7) |  |  |
| CKD |  |  |  |  |  | χ² test | 0.020 |
| No, n(%) | 353 (74.0) | 93 (77.5) | 98 (82.4) | 83 (69.7) | 79 (66.4) |  |  |
|  |  |  |  |  |  |  |  |
| Yes, n(%) | 124 (26.0) | 27 (22.5) | 21 (17.6) | 36 (30.3) | 40 (33.6) |  |  |
| HF |  |  |  |  |  | χ² test | 0.062 |
| HFrEF, n(%) | 168 (35.2) | 40 (33.3) | 38 (31.9) | 39 (32.8) | 51 (42.9) |  |  |
|  |  |  |  |  |  |  |  |
| HFmrEF, n(%) | 78 (16.4) | 17 (14.2) | 20 (16.8) | 29 (24.4) | 12 (10.1) |  |  |
| HFpEF, n(%) | 231 (48.4) | 63 (52.5) | 61 (51.3) | 51 (42.9) | 56 (47.1) |  |  |
| NYHA |  |  |  |  |  | χ² test | 0.003 |
| NYHA II, n(%) | 129 (27.0) | 38 (31.7) | 40 (33.6) | 27 (22.7) | 24 (20.2) |  |  |
|  |  |  |  |  |  |  |  |
| NYHA III, n(%) | 249 (52.2) | 65 (54.2) | 64 (53.8) | 62 (52.1) | 58 (48.7) |  |  |
| NYHA IV, n(%) | 99 (20.8) | 17 (14.2) | 15 (12.6) | 30 (25.2) | 37 (31.1) |  |  |
| MI |  |  |  |  |  | χ² test | 0.866 |
| No, n(%) | 398 (83.4) | 98 (81.7) | 99 (83.2) | 102 (85.7) | 99 (83.2) |  |  |
|  |  |  |  |  |  |  |  |
| Yes, n(%) | 79 (16.6) | 22 (18.3) | 20 (16.8) | 17 (14.3) | 20 (16.8) |  |  |
| PCI |  |  |  |  |  | χ² test | 0.574 |
| No, n(%) | 347 (72.7) | 84 (70.0) | 84 (70.6) | 92 (77.3) | 87 (73.1) |  |  |
|  |  |  |  |  |  |  |  |
| Yes, n(%) | 130 (27.3) | 36 (30.0) | 35 (29.4) | 27 (22.7) | 32 (26.9) |  |  |
| Diabetes |  |  |  |  |  | χ² test | 1.000 |
| Yes, n(%) | 477 (100.0) | 120 (100.0) | 119 (100.0) | 119 (100.0) | 119 (100.0) |  |  |
| Antidiabetic Agents |  |  |  |  |  | χ² test | 0.624 |
| No, n(%) | 70 (14.7) | 20 (16.7) | 20 (16.8) | 14 (11.8) | 16 (13.4) |  |  |
|  |  |  |  |  |  |  |  |
| Yes, n(%) | 407 (85.3) | 100 (83.3) | 99 (83.2) | 105 (88.2) | 103 (86.6) |  |  |
| Antihypertensive Agents |  |  |  |  |  | χ² test | 0.203 |
| No, n(%) | 120 (25.2) | 35 (29.2) | 34 (28.6) | 29 (24.4) | 22 (18.5) |  |  |
|  |  |  |  |  |  |  |  |
| Yes, n(%) | 357 (74.8) | 85 (70.8) | 85 (71.4) | 90 (75.6) | 97 (81.5) |  |  |
| Hospital |  |  |  |  |  | χ² test | 0.445 |
| Zhejiang Provincial Hospital of Chinese Medicine, n(%) | 223 (46.8) | 51 (42.5) | 52 (43.7) | 59 (49.6) | 61 (51.3) |  |  |
|  |  |  |  |  |  |  |  |
| Zhejiang Xinhua Hospital, n(%) | 254 (53.2) | 69 (57.5) | 67 (56.3) | 60 (50.4) | 58 (48.7) |  |  |
| 18-month MACE |  |  |  |  |  | χ² test | <0.001 |
| Non-MACE, n(%) | 286 (60.0) | 100 (83.3) | 101 (84.9) | 56 (47.1) | 29 (24.4) |  |  |
|  |  |  |  |  |  |  |  |
| With MACE, n(%) | 191 (40.0) | 20 (16.7) | 18 (15.1) | 63 (52.9) | 90 (75.6) |  |  |

SPISE-IR, simple proxy insulin resistance index; MACE, major adverse cardiovascular events; WBC, white blood cell count; LVEF, left ventricular ejection fraction; LVDD, left ventricular end-diastolic diameter; HbA1c, glycated hemoglobin; FPG, fasting plasma glucose; TC, total cholesterol; LDL-C, low-density lipoprotein cholesterol; TG, triglyceride; HDL-C, high-density lipoprotein cholesterol; Cr, serum creatinine; LDH, lactate dehydrogenase; ALB, albumin; ALT, alanine aminotransferase; AST, aspartate aminotransferase; eGFR, estimated glomerular filtration rate; CK-MB, creatine kinase-MB isoenzyme; NT-proBNP, N-terminal pro-B-type natriuretic peptide; TnI, cardiac troponin I; RBC, red blood cell count; PLT, platelet count; ANC, absolute neutrophil count; NEUT, neutrophil percentage; CRP, C-reactive protein; BMI, body mass index; CKD, chronic kidney disease; HF, heart failure; HFrEF, heart failure with reduced ejection fraction; HFmrEF, heart failure with mildly reduced ejection fraction; HFpEF, heart failure with preserved ejection fraction; NYHA, New York Heart Association cardiac function classification; MI, myocardial infarction; PCI, percutaneous coronary intervention.
