# Supplementary material for: Study on the correlation between SPISE-IR, METs-IR, TyG and short-term adverse cardiovascular events in patients with heart failure and diabetes mellitus
Source: Front Nutr. 2026 Oct 16;13:1938459. doi: 10.3389/fnut.2026.1938459 (PMC13719381; doi:10.3389/fnut.2026.1938459)
Supplement: Supplementary file 3 [file Table_3.docx]

**Supplementary Table 3. Baseline characteristics grouped by METS-IR quartiles**

| **Variables** | **Total (n = 477)** | **Q1 (n = 120)** | **Q2 (n = 119)** | **Q3 (n = 119)** | **Q4 (n = 119)** | **Statistic** | **P** |
| --- | --- | --- | --- | --- | --- | --- | --- |
| Age(years) | 78.00 (70.00, 85.00) | 78.00 (72.00, 84.00) | 77.00 (70.00, 83.00) | 79.00 (69.00, 85.00) | 78.00 (71.00, 86.00) | Kruskal-Wallis | 0.646 |
| LVEF (%) | 57.00 (49.00, 64.00) | 59.00 (50.00, 66.00) | 57.00 (48.00, 65.00) | 56.00 (48.00, 62.20) | 57.00 (51.00, 62.00) | Kruskal-Wallis | 0.125 |
| LVDD(mm) | 50.00 (46.00, 54.00) | 48.70 (43.77, 53.00) | 49.00 (46.00, 53.00) | 51.00 (47.00, 55.00) | 50.00 (46.85, 55.50) | Kruskal-Wallis | 0.006 |
| HbA1c(%) | 6.90 (6.30, 7.80) | 6.80 (6.10, 7.53) | 7.10 (6.30, 7.80) | 6.90 (6.30, 7.75) | 6.90 (6.20, 8.20) | Kruskal-Wallis | 0.441 |
| FPG(mmol/L) | 6.72 (4.81, 9.58) | 5.83 (4.81, 8.26) | 6.83 (5.26, 8.78) | 6.22 (3.97, 9.05) | 8.50 (5.47, 13.73) | Kruskal-Wallis | <0.001 |
| TC(mmol/L) | 3.79 (3.06, 4.54) | 3.96 (3.28, 4.49) | 3.95 (3.10, 4.80) | 3.65 (2.90, 4.58) | 3.58 (2.79, 4.45) | Kruskal-Wallis | 0.237 |
| LDL-C(mmol/L) | 1.77 (1.32, 2.45) | 1.77 (1.37, 2.38) | 1.80 (1.38, 2.56) | 1.77 (1.31, 2.58) | 1.77 (1.28, 2.47) | Kruskal-Wallis | 0.907 |
| TG(mmol/L) | 1.12 (0.76, 1.89) | 0.99 (0.76, 1.46) | 1.06 (0.83, 1.54) | 1.28 (0.80, 2.21) | 1.21 (0.67, 2.47) | Kruskal-Wallis | 0.056 |
| HDL-C(mmol/L) | 0.97 (0.57, 1.53) | 1.29 (0.70, 1.72) | 1.06 (0.75, 1.31) | 0.69 (0.50, 1.07) | 0.82 (0.47, 1.79) | Kruskal-Wallis | <0.001 |
| Cr(μmol/L) | 90.00 (69.80, 125.00) | 84.20 (65.00, 114.30) | 87.70 (70.40, 108.50) | 91.40 (71.15, 122.25) | 111.00 (79.05, 142.40) | Kruskal-Wallis | <0.001 |
| LDH(U/L) | 197.00 (165.00, 237.00) | 190.50 (158.50, 254.75) | 197.00 (168.50, 237.50) | 201.00 (164.00, 233.50) | 198.00 (174.00, 232.00) | Kruskal-Wallis | 0.775 |
| ALB(g/L) | 34.80 (31.70, 38.10) | 35.05 (32.50, 38.62) | 34.80 (31.70, 37.90) | 35.30 (32.35, 38.20) | 33.60 (30.85, 37.00) | Kruskal-Wallis | 0.019 |
| ALT(U/L) | 18.00 (12.00, 29.00) | 18.00 (14.00, 28.00) | 17.00 (12.00, 28.00) | 19.00 (12.00, 27.50) | 17.00 (12.00, 33.50) | Kruskal-Wallis | 0.972 |
| AST(U/L) | 22.00 (17.00, 31.00) | 23.00 (19.00, 31.00) | 24.00 (17.00, 31.00) | 21.00 (17.00, 29.00) | 22.00 (17.00, 31.50) | Kruskal-Wallis | 0.635 |
| eGFR, (mL·min⁻¹·1.73m⁻²) | 66.20 (47.40, 90.84) | 72.71 (52.98, 97.55) | 73.30 (50.91, 89.41) | 67.30 (46.87, 90.05) | 54.20 (40.04, 74.90) | Kruskal-Wallis | <0.001 |
| CK-MB(U/L) | 12.60 (6.58, 18.20) | 11.05 (5.05, 17.75) | 13.10 (8.65, 18.45) | 11.00 (6.50, 16.95) | 13.70 (8.80, 19.00) | Kruskal-Wallis | 0.026 |
| NT-proBNP(pg/mL) | 2387.00 (1238.00, 5364.00) | 2189.50 (966.75, 4213.75) | 2113.00 (1211.00, 3867.00) | 2271.00 (1359.00, 4699.00) | 3655.00 (1542.50, 7801.50) | Kruskal-Wallis | 0.001 |
| TnI(μg/L) | 0.02 (0.01, 0.06) | 0.02 (0.01, 0.06) | 0.02 (0.01, 0.06) | 0.02 (0.01, 0.05) | 0.02 (0.01, 0.06) | Kruskal-Wallis | 0.668 |
| RBC, ×10¹²/L | 4.03 ± 0.76 | 4.00 ± 0.66 | 4.08 ± 0.74 | 4.09 ± 0.78 | 3.96 ± 0.87 | Welch ANOVA | 0.569 |
| WBC, ×10⁹/L | 6.60 (5.10, 8.20) | 7.00 (5.18, 8.22) | 6.50 (5.20, 8.20) | 6.50 (5.00, 8.45) | 6.60 (5.20, 8.00) | Kruskal-Wallis | 0.852 |
| PLT, ×10⁹/L | 175.00 (139.00, 218.00) | 168.00 (139.75, 215.25) | 181.00 (149.00, 220.50) | 185.00 (140.50, 228.50) | 164.00 (129.50, 198.00) | Kruskal-Wallis | 0.068 |
| ANC, ×10⁹/L | 4.40 (3.20, 6.00) | 4.60 (3.38, 6.15) | 4.30 (3.35, 5.90) | 4.20 (3.10, 6.20) | 4.50 (3.20, 5.90) | Kruskal-Wallis | 0.859 |
| NEUT(%) | 69.23 (60.40, 76.90) | 71.30 (60.94, 77.23) | 67.44 (60.10, 75.55) | 67.60 (59.23, 77.15) | 69.80 (60.51, 76.32) | Kruskal-Wallis | 0.612 |
| CRP(mg/L) | 5.00 (1.70, 16.28) | 4.09 (1.35, 16.45) | 5.10 (1.70, 13.50) | 4.92 (1.92, 15.37) | 6.16 (2.04, 19.92) | Kruskal-Wallis | 0.583 |
| Height(cm) | 163.00 (156.00, 168.00) | 159.00 (155.00, 167.00) | 165.00 (158.00, 170.00) | 165.00 (157.00, 170.00) | 162.00 (156.00, 167.00) | Kruskal-Wallis | 0.002 |
| Weight(kg) | 64.70 (58.00, 72.00) | 53.00 (49.00, 60.00) | 64.00 (58.00, 70.00) | 67.00 (62.00, 73.00) | 72.00 (65.50, 79.00) | Kruskal-Wallis | <0.001 |
| BMI | 23.64 (21.89, 25.58) | 21.67 (17.09, 23.68) | 24.01 (22.71, 24.98) | 23.40 (21.83, 25.43) | 29.69 (23.28, 35.54) | Kruskal-Wallis | <0.001 |
| SPISE | 8.18 (7.13, 9.27) | 10.35 (9.47, 11.59) | 8.52 (8.15, 8.90) | 7.73 (7.28, 8.20) | 6.24 (5.59, 6.89) | Kruskal-Wallis | <0.001 |
| SPISE-IR | 1.22 (1.08, 1.40) | 0.97 (0.86, 1.06) | 1.17 (1.12, 1.23) | 1.29 (1.22, 1.37) | 1.60 (1.45, 1.79) | Kruskal-Wallis | <0.001 |
| METS-IR | 39.99 (36.28, 44.73) | 32.65 (30.12, 34.68) | 38.47 (37.57, 39.42) | 41.81 (40.84, 43.09) | 49.65 (46.59, 54.23) | Kruskal-Wallis | <0.001 |
| TyG | 8.76 (8.34, 9.22) | 8.50 (8.13, 8.91) | 8.74 (8.35, 9.08) | 8.77 (8.42, 9.25) | 9.01 (8.65, 9.58) | Kruskal-Wallis | <0.001 |
| Gender |  |  |  |  |  | χ² test | 0.059 |
| Male, n(%) | 250 (52.4) | 54 (45.0) | 67 (56.3) | 72 (60.5) | 57 (47.9) |  |  |
| Female, n(%) | 227 (47.6) | 66 (55.0) | 52 (43.7) | 47 (39.5) | 62 (52.1) |  |  |
| Smoking |  |  |  |  |  | χ² test | 0.947 |
| No, n(%) | 327 (68.6) | 83 (69.2) | 83 (69.7) | 82 (68.9) | 79 (66.4) |  |  |
| Yes, n(%) | 150 (31.4) | 37 (30.8) | 36 (30.3) | 37 (31.1) | 40 (33.6) |  |  |
| Hypertension |  |  |  |  |  | χ² test | 0.047 |
| No, n(%) | 103 (21.6) | 31 (25.8) | 33 (27.7) | 21 (17.6) | 18 (15.1) |  |  |
| Yes, n(%) | 374 (78.4) | 89 (74.2) | 86 (72.3) | 98 (82.4) | 101 (84.9) |  |  |
| CKD |  |  |  |  |  | χ² test | 0.007 |
| No, n(%) | 353 (74.0) | 92 (76.7) | 96 (80.7) | 91 (76.5) | 74 (62.2) |  |  |
| Yes, n(%) | 124 (26.0) | 28 (23.3) | 23 (19.3) | 28 (23.5) | 45 (37.8) |  |  |
| HF |  |  |  |  |  | χ² test | 0.021 |
| HFrEF, n(%) | 168 (35.2) | 41 (34.2) | 42 (35.3) | 32 (26.9) | 53 (44.5) |  |  |
| HFmrEF, n(%) | 78 (16.4) | 16 (13.3) | 23 (19.3) | 28 (23.5) | 11 (9.2) |  |  |
| HFpEF, n(%) | 231 (48.4) | 63 (52.5) | 54 (45.4) | 59 (49.6) | 55 (46.2) |  |  |
| NYHA |  |  |  |  |  | χ² test | 0.002 |
| NYHA II, n(%) | 129 (27.0) | 39 (32.5) | 38 (31.9) | 26 (21.8) | 26 (21.8) |  |  |
| NYHA III, n(%) | 249 (52.2) | 70 (58.3) | 59 (49.6) | 64 (53.8) | 56 (47.1) |  |  |
| NYHA IV, n(%) | 99 (20.8) | 11 (9.2) | 22 (18.5) | 29 (24.4) | 37 (31.1) |  |  |
| MI |  |  |  |  |  | χ² test | 0.837 |
| No, n(%) | 398 (83.4) | 98 (81.7) | 98 (82.4) | 102 (85.7) | 100 (84.0) |  |  |
| Yes, n(%) | 79 (16.6) | 22 (18.3) | 21 (17.6) | 17 (14.3) | 19 (16.0) |  |  |
| PCI |  |  |  |  |  | χ² test | 0.385 |
| No, n(%) | 347 (72.7) | 82 (68.3) | 85 (71.4) | 93 (78.2) | 87 (73.1) |  |  |
| Yes, n(%) | 130 (27.3) | 38 (31.7) | 34 (28.6) | 26 (21.8) | 32 (26.9) |  |  |
| Diabetes |  |  |  |  |  | χ² test | 1.000 |
| Yes, n(%) | 477 (100.0) | 120 (100.0) | 119 (100.0) | 119 (100.0) | 119 (100.0) |  |  |
| Antidiabetic Agents |  |  |  |  |  | χ² test | 0.356 |
| No, n(%) | 70 (14.7) | 20 (16.7) | 21 (17.6) | 17 (14.3) | 12 (10.1) |  |  |
| Yes, n(%) | 407 (85.3) | 100 (83.3) | 98 (82.4) | 102 (85.7) | 107 (89.9) |  |  |
| Antihypertensive Agents |  |  |  |  |  | χ² test | 0.164 |
| No, n(%) | 120 (25.2) | 33 (27.5) | 37 (31.1) | 27 (22.7) | 23 (19.3) |  |  |
| Yes, n(%) | 357 (74.8) | 87 (72.5) | 82 (68.9) | 92 (77.3) | 96 (80.7) |  |  |
| Hospital |  |  |  |  |  | χ² test | 0.881 |
| Zhejiang Provincial Hospital of Chinese Medicine, n(%) | 223 (46.8) | 57 (47.5) | 52 (43.7) | 58 (48.7) | 56 (47.1) |  |  |
| Zhejiang Xinhua Hospital, n(%) | 254 (53.2) | 63 (52.5) | 67 (56.3) | 61 (51.3) | 63 (52.9) |  |  |
| 18-month MACE |  |  |  |  |  | χ² test | <0.001 |
| Non-MACE, n(%) | 286 (60.0) | 104 (86.7) | 95 (79.8) | 56 (47.1) | 31 (26.1) |  |  |
| With MACE, n(%) | 191 (40.0) | 16 (13.3) | 24 (20.2) | 63 (52.9) | 88 (73.9) |  |  |

METs-IR, metabolic insulin resistance index; MACE, major adverse cardiovascular events; WBC, white blood cell count; LVEF, left ventricular ejection fraction; LVDD, left ventricular end-diastolic diameter; HbA1c, glycated hemoglobin; FPG, fasting plasma glucose; TC, total cholesterol; LDL-C, low-density lipoprotein cholesterol; TG, triglyceride; HDL-C, high-density lipoprotein cholesterol; Cr, serum creatinine; LDH, lactate dehydrogenase; ALB, albumin; ALT, alanine aminotransferase; AST, aspartate aminotransferase; eGFR, estimated glomerular filtration rate; CK-MB, creatine kinase-MB isoenzyme; NT-proBNP, N-terminal pro-B-type natriuretic peptide; TnI, cardiac troponin I; RBC, red blood cell count; PLT, platelet count; ANC, absolute neutrophil count; NEUT, neutrophil percentage; CRP, C-reactive protein; BMI, body mass index; CKD, chronic kidney disease; HF, heart failure; HFrEF, heart failure with reduced ejection fraction; HFmrEF, heart failure with mildly reduced ejection fraction; HFpEF, heart failure with preserved ejection fraction; NYHA, New York Heart Association cardiac function classification; MI, myocardial infarction; PCI, percutaneous coronary intervention.
