# Supplementary material for: Study on the correlation between SPISE-IR, METs-IR, TyG and short-term adverse cardiovascular events in patients with heart failure and diabetes mellitus
Source: Front Nutr. 2026 Oct 16;13:1938459. doi: 10.3389/fnut.2026.1938459 (PMC13719381; doi:10.3389/fnut.2026.1938459)
Supplement: Supplementary file 4 [file Table_4.docx]

**Supplementary Table S4.Multicollinearity analysis**

| **Variable** | **GVIF** | **Df** | **GVIF^(1/(2*Df))** |
| --- | --- | --- | --- |
| Gender | 3.403 | 1 | 1.845 |
| Smoking | 1.846 | 1 | 1.359 |
| Hypertension | 5.571 | 1 | 2.36 |
| HF classification | 1.917 | 2 | 1.177 |
| CKD | 1.672 | 1 | 1.293 |
| NYHA | 1.499 | 2 | 1.107 |
| MI | 2.033 | 1 | 1.426 |
| PCI | 1.922 | 1 | 1.386 |
| AntihypertensiveAgents | 5.886 | 1 | 2.426 |
| AntidiabeticAgents | 1.202 | 1 | 1.096 |
| Age | 1.977 | 1 | 1.406 |
| LVEF | 2.262 | 1 | 1.504 |
| LVDD | 1.936 | 1 | 1.391 |
| HbA1c | 1.47 | 1 | 1.212 |
| FPG | 6.485 | 1 | 2.546 |
| TC | 12.738 | 1 | 3.569 |
| LDL-C | 11.758 | 1 | 3.429 |
| TG | 7.995 | 1 | 2.828 |
| HDL-C | 5.579 | 1 | 2.362 |
| Cr | 2.725 | 1 | 1.651 |
| LDH | 5.609 | 1 | 2.368 |
| ALB | 1.41 | 1 | 1.187 |
| ALT | 15.924 | 1 | 3.99 |
| AST | 19.932 | 1 | 4.464 |
| eGFR | 2.558 | 1 | 1.599 |
| CK-MB | 1.642 | 1 | 1.281 |
| NT-proBNP | 1.561 | 1 | 1.249 |
| Tnl | 1.934 | 1 | 1.391 |
| RBC | 1.887 | 1 | 1.374 |
| WBC | 29.559 | 1 | 5.437 |
| PLT | 1.431 | 1 | 1.196 |
| ANC | 40.474 | 1 | 6.362 |
| NEUT | 4.976 | 1 | 2.231 |
| CRP | 1.413 | 1 | 1.189 |
| Height | 65.33 | 1 | 8.083 |
| Weight | 172.243 | 1 | 13.124 |
| BMI | 136.682 | 1 | 11.691 |
| SPISE-IR | 62.08 | 1 | 7.879 |
| METS-IR | 21.005 | 1 | 4.583 |
| TyG | 12.3 | 1 | 3.507 |

GVIF, generalized variance inflation factor; Df, degree of freedom; HF, heart failure; CKD, chronic kidney disease; NYHA, New York Heart Association cardiac function classification; MI, myocardial infarction; PCI, percutaneous coronary intervention; LVEF, left ventricular ejection fraction; LVDD, left ventricular end-diastolic diameter; HbA1c, glycated hemoglobin; FPG, fasting plasma glucose; TC, total cholesterol; LDL-C, low-density lipoprotein cholesterol; TG, triglyceride; HDL-C, high-density lipoprotein cholesterol; Cr, serum creatinine; LDH, lactate dehydrogenase; ALB, albumin; ALT, alanine aminotransferase; AST, aspartate aminotransferase; eGFR, estimated glomerular filtration rate; CK-MB, creatine kinase-MB isoenzyme; NT-proBNP, N-terminal pro-B-type natriuretic peptide; TnI, cardiac troponin I; RBC, red blood cell count; WBC, white blood cell count; PLT, platelet count; ANC, absolute neutrophil count; NEUT, neutrophil percentage; CRP, C-reactive protein; BMI, body mass index; SPISE-IR, simple proxy insulin resistance index; METs-IR, metabolic insulin resistance index; TyG, triglyceride-glucose index.
